# Porous Silicon Nanocarriers Boost the Immunomodulation of Mitochondria-Targeted Bovine Serum Albumins on Macrophage Polarization

**DOI:** 10.1021/acsnano.2c07439

**Published:** 2023-01-04

**Authors:** Jialiang Li, Jiqiang Fan, Yan Gao, Shuodan Huang, Di Huang, Jiachen Li, Xiaoyu Wang, Hélder A. Santos, Pingping Shen, Bing Xia

**Affiliations:** † College of Science, Nanjing Forestry University, Nanjing210037, China; ‡ Department of Geriatric Medicine, The Second Affiliated Hospital and Yuying Children’s Hospital of Wenzhou Medical University, Wenzhou325027, China; § State Key Laboratory of Pharmaceutical Biotechnology and The Comprehensive Cancer Center, Nanjing Drum Tower Hospital, The Affiliated Hospital of Nanjing University Medical School, Nanjing University, Nanjing210023, China; ∥ Department of Biomedical Engineering, University Medical Center Groningen, University of Groningen, Antonius Deusinglaan 1, 9713 AVGroningen, The Netherlands; ⊥ W. J. Kolff Institute for Biomedical Engineering and Materials Science, University Medical Center Groningen, University of Groningen, Antonius Deusinglaan 1, 9713 AVGroningen, The Netherlands

**Keywords:** porous silicon nanoparticles, albumin proteins, mitochondrial targeting, reactive oxygen species (ROS), macrophage polarization, signaling transduction pathways

## Abstract

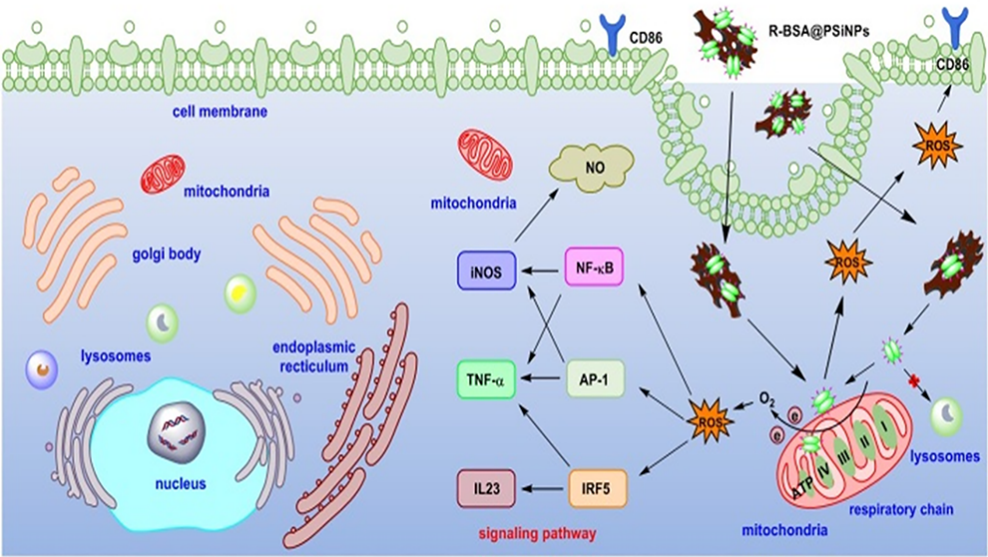

The development of nanosystems with intrinsic immunomodulatory effects on macrophage polarization is important for the macrophage-targeted immunotherapy. Here, mitochondria-targeted bovine serum albumins (BSAs) via the conjugation of fluorescent, lipophilic, and cationic rhodamine 110 molecules can efficiently enhance the gene expression of the proinflammatory phenotype of macrophages and correspondingly inhibit the gene expression of their anti-inflammatory phenotype. On this basis, porous silicon nanocarriers can further boost the immunomodulation of these mitochondria-targeted BSAs *in vitro* or *in vivo*, accompanied by the secretion of proinflammatory mediators including tumor necrosis factor α, nitric oxide, and reactive oxygen species (ROS). Meanwhile, BSA coatings can also improve the biocompatibility of porous silicon nanoparticulate cores on macrophages. Finally, the mechanism investigations demonstrate that porous silicon nanocarriers can efficiently deliver mitochondria-targeted BSA into macrophages to generate mitochondrial ROS via the interference with mitochondrial respiratory chains, which can further trigger the downstream signaling transduction pathways for the proinflammatory transition. Considering the good biosafety and versatile loading capability, this developed porous silicon@BSA nanosystem with a strong proinflmmatory regulatory effect has important potential on the combinatorial chemoimmunotherapy against cancer or viral/bacterial-related infectious diseases.

Macrophages as innate immune cells can execute diverse and dynamic immune functions to mediate the defense and homeostasis of the bodies, the development of diseases, or tissue regeneration and remodeling.^1^ Under healthy or pathological conditions, macrophages with good plasticity can be polarized into two distinct phenotypes, such as classically activated proinflammatory (M1) or alternatively activated anti-inflammatory phenotypes (M2).^2^ Except for the balance between M1/M2 polarization of macrophages necessary for healthy homeostasis, M2 polarization of macrophages is important for tissue regeneration or inflammation resolution, or M1 phenotype facilitates the antiviral, antimicrobial, or antitumoral activities.^3,4^ Macrophage M1 polarization can induce the secretion of proinflammatory cytokines (e.g., tumor necrosis factor α (TNF-α), interleukin 1β (IL-1β), IL-6, or IL-12, etc.) or chemokines (e.g., CC chemokine ligand 2 (CCL2) or CXC chemokine ligand 1 (CXCL-1), etc.), and also produce reactive oxygen or reactive nitrogen species (ROS and RNOS). These proinflammatory cytokines ROS or RNOS can not only directly kill viruses, microbes, or tumor cells but also efficiently reverse the immunosuppressive microenvironments of disease sites.^5,6^ Moreover, the activation of macrophages as innate immune cells is the first step to prime the body immunity, which can successively present antigens and promote the differentiation of T helper 1 lymphocytes to produce more proinflammatory cytokines and chemokines.^7,8^ Finally, membranes, exosomes, or whole cells derived of M1 macrophages can also be used as drug carriers to pass through biological barriers including brain–blood barriers and deliver therapeutic agents to inflammation sites.^9,10^ Therefore, the mediation of macrophage M1 polarization plays a critical role in the immunotherapy of cancer, viral-related, or bacterial-related infectious diseases. The polarization of macrophage is closely related to multiple physical, chemical, or biological cues.^11,12^ For instance, a classic strategy is the usage of lipopolysaccharide (an endotoxin, LPS) and interferon γ (IFN-γ) to activate macrophage M1 polarization. However, LPS molecules inevitably cause systemic inflammatory syndromes in the body, such as sepsis, hypotension, or shock.^13^ Additionally, clinical and preclinical small molecule drugs for the macrophage immunomodulation remain limited, due to their off-target toxicities throughout the body.^14^ In contrast, nanocarriers modified with specific ligands can efficiently deliver immunomodulatory agents into macrophages at lower dose to efficiently improve their biosafety.^14^ Moreover, the intrinsic modulatory functions of nanovectors can also drive macrophage phenotype for M1/M2 transition, which is beneficial for the combined chemoimmunotherapy.^15−19^

Among various inorganic nanoparticles, porous silicon nanoparticles (PSiNPs) with good biocompatibility and biodegradability have been extensively applied on the diagnosis and therapy of diseases, especially to activate the innate immunity for the immunotherapy.^20−23^ For example, PSiNPs-based nanocarriers were reported to deliver albumin-bound paclitaxel, which can promote the migration and proinflammatory polarization of macrophages for higher antitumoral efficacy.^24^ PSiNPs can also introduce small interfering RNAs (siRNA) into macrophages to inhibit their proinflammatory shifting against bacterial infections^25^ or reprogram tumor-associated macrophages into a proinflammatory state to suppress the growth of tumors.^26^ In addition, our previous studies demonstrated that PSiNPs had intrinsic regulatory effect to activate innate immune cells, such as macrophages or dendritic cells, mainly dependent on their size and surface properties.^27,28^ For instance, in comparison to oxidized PSiNPs with hydrophilic surfaces and negative ζ-potential, hydrogen or alkyl-terminated PSiNPs with hydrophobic surfaces, or amine-terminated PSiNPs with positive charges can promote the secretion of ROS, RNOS, and TNF-α of macrophages.^28^ Due to the advantages of rich reactive groups, good biocompatibility, and low-cost industrial production, albumins derived of plasma or serum have been widely applied as nanocarriers to deliver therapeutic agents in the clinic, which has been approved by Food and Drug Administration (FDA).^29^ Notably, it was also found that the glycosylation, maleylation, or acetylation of bovine serum albumins (BSAs) can induce the proinflammatory polarization of macrophages, accompanied by nitric oxide (NO) generation or TNF-α secretion.^30−34^ Herein, BSAs were conjugated with fluorescent, lipophilic, and cationic rhodamine 110 molecules to prepare mitochondria-targeted R-BSAs and subsequently attached onto PSiNPs via hydrophobic interactions to obtain R-BSA@PSiNPs nanocomposites, as illustrated in Schemes 1 and S1. Furthermore, *in vitro* and *in vivo* results demonstrate that the regulatory effect of R-BSA on macrophage M1 polarization can be augmented by PSiNPs nanocarriers. Meanwhile, R-BSA coatings can also improve the biocompatibility of PSiNPs cores on macrophages. Finally, the mechanism studies indicate that macrophage M1 polarization is mainly determined by mitochondria-controlling ROS generation.

**Scheme 1:**
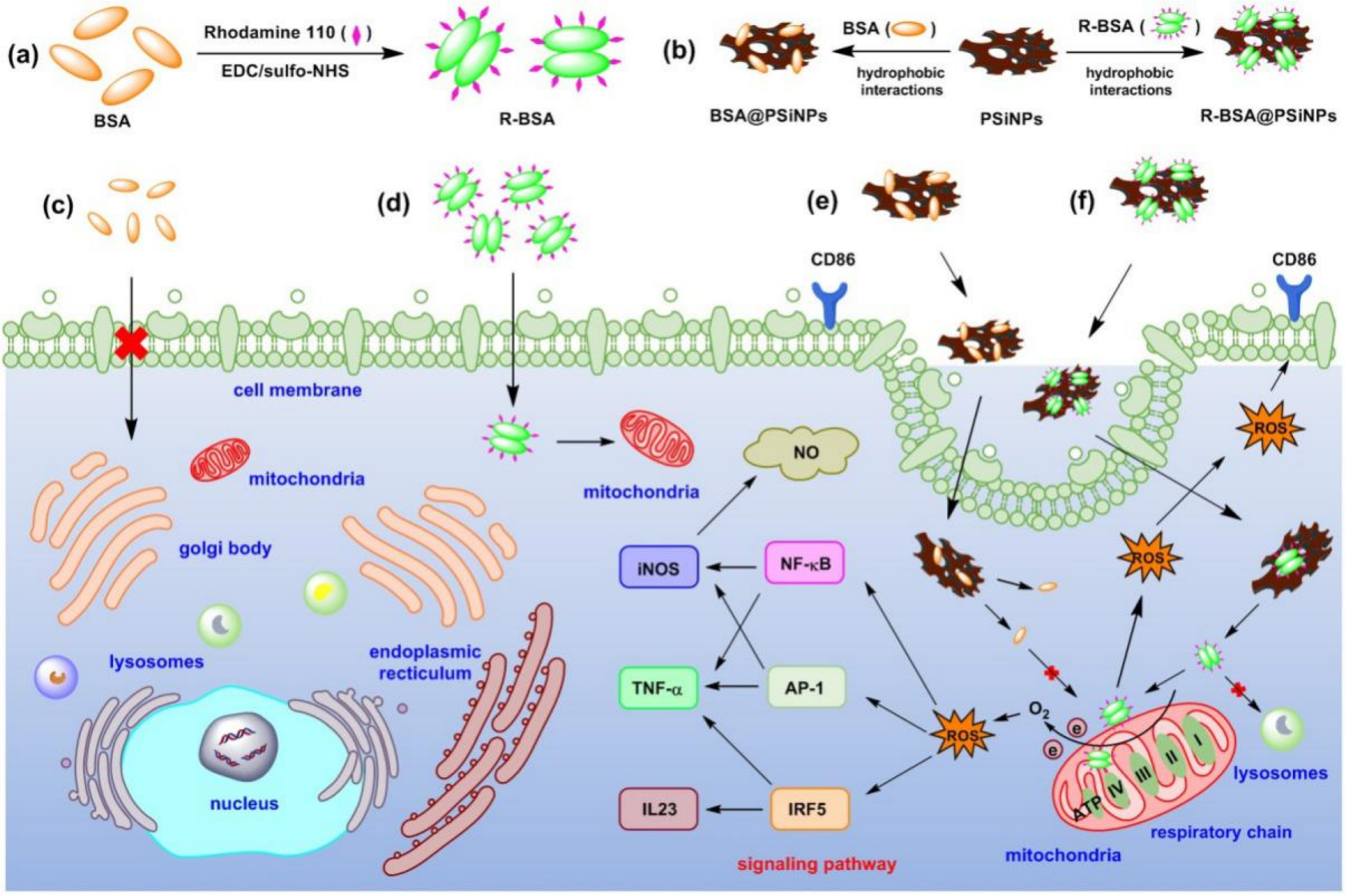
Synthesis Route of (a) R-BSA, (b) BSA@PSiNPs, R-BSA@PSiNPs Nanocomposites and Schematic Illustration of the Interactions between Macrophages and (c) BSA, (d) R-BSA, (e) BSA@PSiNPs, and (f) R-BSA@PSiNPs, and the Corresponding Signaling Pathways

## Results and Discussion

### Regulatory Effect of R-BSA on Macrophage Polarization

As seen in Schemes 1a and S1, BSA can be covalently bound with fluorescent, lipophilic, and cationic rhodamine 110 molecules via 1-ethyl-3-(3-dimethylaminopropyl)carbodiimide hydrochloride (EDC) and *N*-hydroxysulfosuccinimide sodium salt (sulfo-NHS) reaction to obtain R-BSA.^35^ And as-prepared R-BSA samples were further characterized by dynamic laser scattering (DLS) measurements, UV–vis spectra, photoluminescence (PL) spectra, and transmission electron microscope (TEM). In Figure S1a,b, compared with naive BSA samples, the appearance of the PL emission peak at 521 nm and UV absorbance peak at 498 nm is due to the conjugation of rhodamine 110 molecules with BSA. In Figure S2a,b, DLS results show that the average hydrodynamic size of naive BSA samples is 6.86 ± 0.47 nm, close to the theoretical model (14 × 4 × 1.4 nm^3^) of a single BSA molecule.^36^ In contrast, the average size of R-BSA increases to 21.56 ± 0.43 nm, indicating the intermolecular cross-linking of BSA molecules, as illustrated in Scheme S1. DLS results also present that the ζ-potential of BSA slightly changes from −9.05 ± 0.55 to −8.09 ± 0.32 mV, with the conjugation of rhodamine 110 molecules containing positive amine moieties. And no changes of their polydispersity index (PDI, 0.26 ± 0.02) indicate that the conjugation of rhodamine 110 molecules has no effect on the aqueous dispersibility of BSA. Moreover, TEM imaging was performed to visualize the morphology of BSA and R-BSA samples. In Figure S2c, the size of naive BSA marked by green dash circles is mostly centered at ∼20 nm, indicating a slight aggregation under ultrahigh vacuum condition of TEM. With the same magnification, the size of R-BSA marked by yellow dash circles increases to ∼40 nm, composed of smaller particles with the size of ∼10 nm (red dash lines) close to single BSA molecule. These TEM results also verify the aggregation of BSA induced by EDC/NHS reaction. Accordingly, these above findings demonstrate that rhodamine 110 molecules have been successfully covalently linked with BSA via EDC/NHS reactions to prepare R-BSA for next experiments.

**Figure 1:**
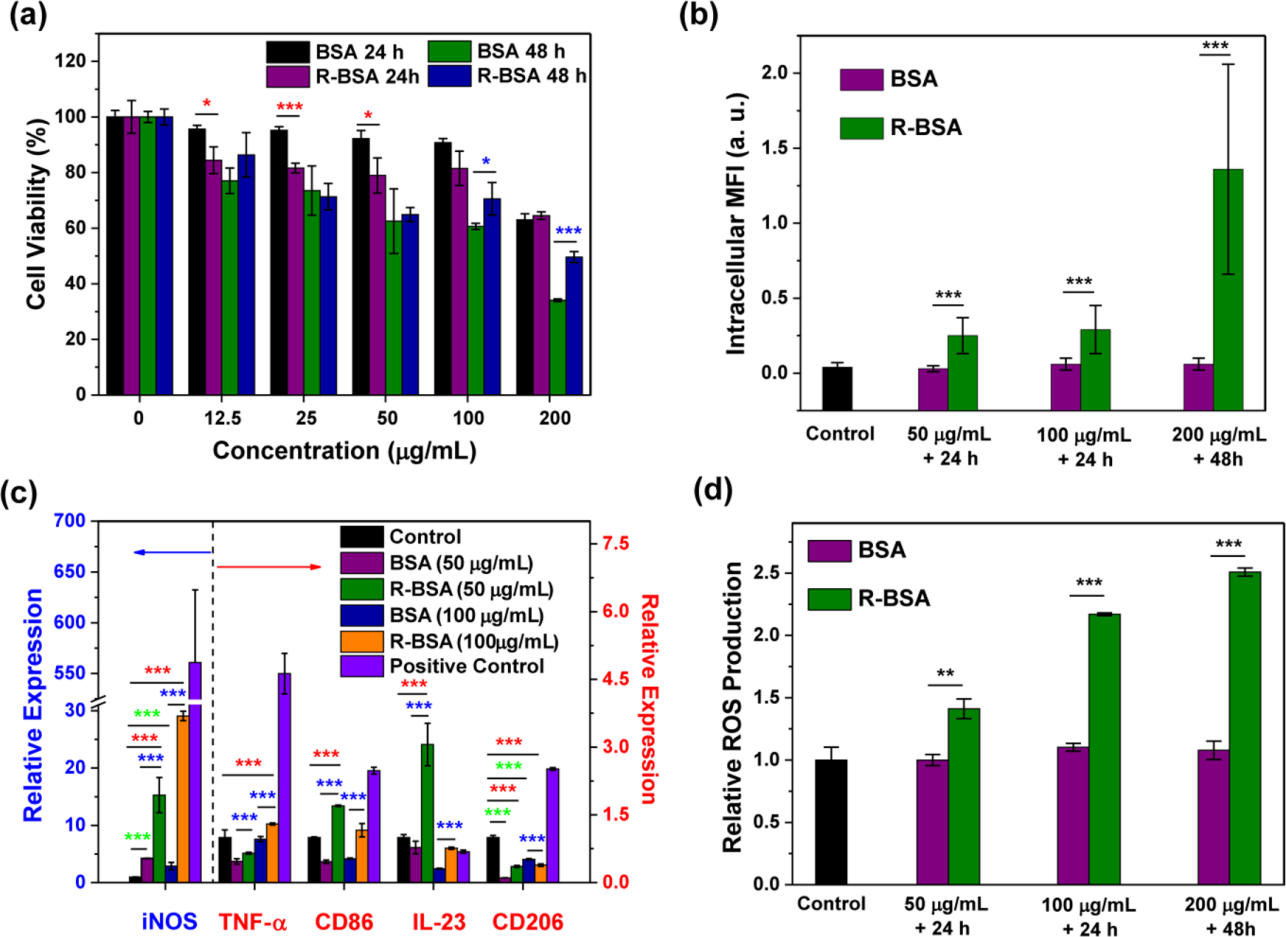
(a) Cell viability of RAW 264.7 cells treated with BSA or R-BSA at the concentration range of 0–200 μg/mL for 24 or 48 h (*n* = 6 biological independent samples); (b) intracellular MFI from ∼100 cells with BSA or R-BSA treatments including 50 μg/mL + 24 h, 100 μg/mL + 24 h, and 200 μg/mL + 48 h; (c) relative gene expression of iNOS, TNF-α, CD86, IL-23, and CD206 in RAW 264.7 cells treated with 50 or 100 μg/mL BSA or R-BSA for 24 h, untreated RAW 264.7 cells as a control, and ones stimulated by LPS + IFN-γ as a positive control (*n* = 3 biological independent samples); and (d) ROS production of RAW 264.7 cells with BSA or R-BSA treatments including 50 μg/mL + 24 h, 100 μg/mL + 24 h, and 200 μg/mL + 48h (*n* = 4 biological independent samples). Error bars are based on standard errors of the mean (***P < 0.001, **P < 0.01, or *P < 0.05 by ANOVA with Tukey’s post-test).

**Figure 2:**
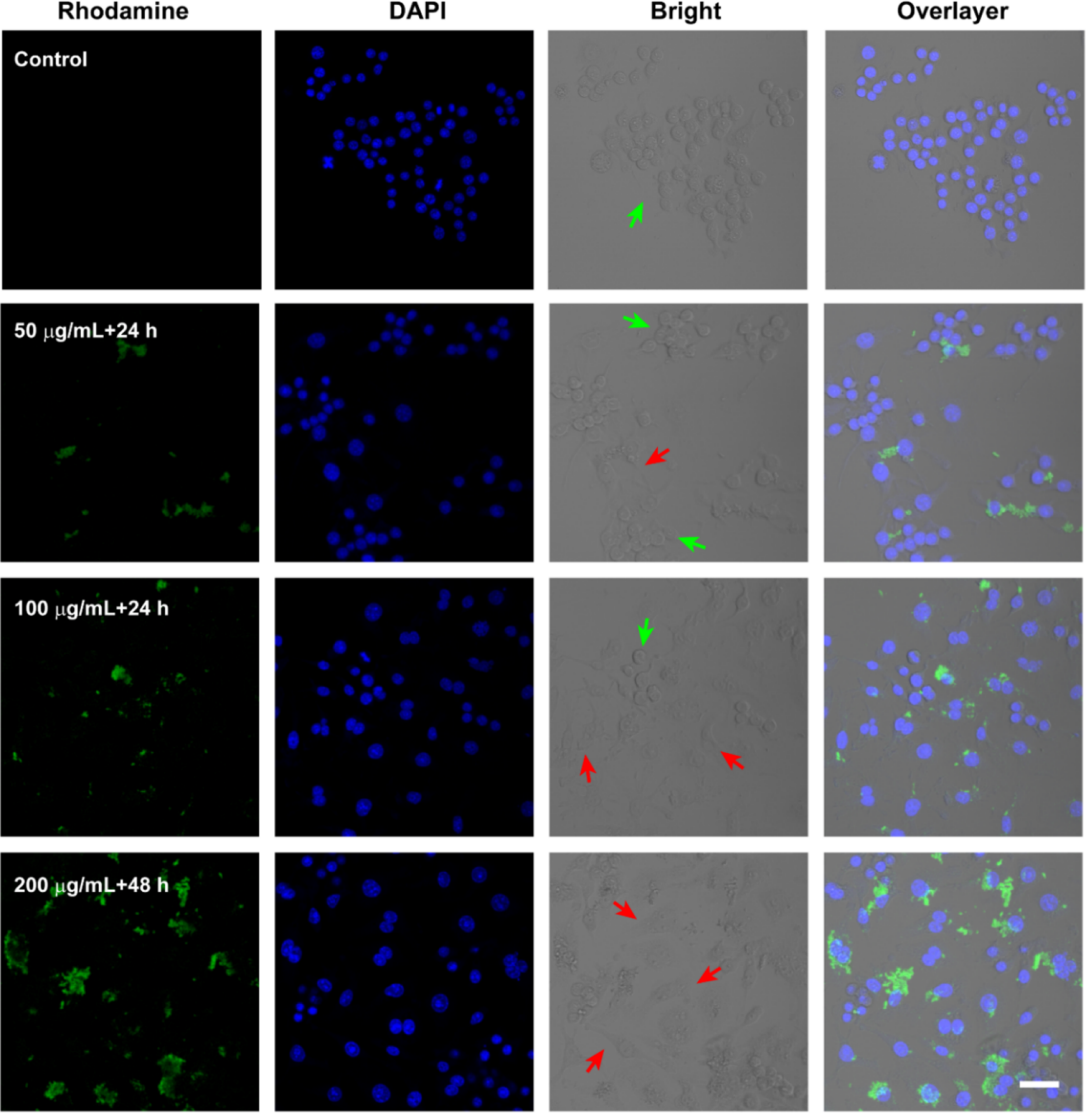
Confocal imaging of RAW 264.7 cells with R-BSA treatments including 50 μg/mL + 24 h, 100 μg/mL + 24 h, and 200 μg/mL + 48h (scale bar = 40 μm).

Moreover, 5 × 10^3^ per mL murine macrophage-like RAW 264.7 cells was incubated with BSA or R-BSA at the concentration range of 0–200 μg/mL for 24 or 48 h to evaluate their cytotoxicity on macrophages by 3-(4,5-dimethylthiazol-2-yl)-2,5-diphenyltetrazolium bromide (MTT) assay. In Figure 1a, MTT results display that under the condition of lower sample concentration, such as 12.5, 25, or 50 μg/mL and shorter incubation time of 24 h, R-BSAs have higher cytotoxicity than naive BSA. Conversely, BSAs show higher cytotoxicity than R-BSAs, under the conditions of higher concentration, such as 100 or 200 μg/mL and longer incubation time of 48 h. For instance, the viability of RAW 264.7 cells treated with 25 μg/mL BSA for 24 h is 95.2 ± 1.3%, higher than 81.6 ± 1.8% of R-BSA with the significant difference of ***P < 0.001 (red asterisks). However, after 48 h incubation with 200 μg/mL BSA, the cell viability is only 34.1 ± 0.5%, much lower than 49.6 ± 1.9% of R-BSA (P*** < 0.001, blue asterisks). In addition, the interactions of BSA or R-BSA with RAW 264.7 cells were monitored by laser scanning confocal microscope (LSCM), recorded in Figure 2. Here, the detectable R-BSA with strong green fluorescence can be directly observed by confocal imaging. However, naive BSA with no intrinsic fluorescence need to be further labeled with fluorescein isothiocyanate (FITC) probes to obtain fluorescent F-BSA, as shown in Scheme S2. And F-BSA was also characterized by UV–vis and PL spectra in Figure S1c,d. Both the UV absorbance peak at 499 nm and the PL emission peak at 522 nm appear in F-BSA, indicating the successful conjugation of FITC probes. In Figure S2a,b, DLS results also display similar hydrodynamic size and ζ-potential of F-BSA with BSA. For confocal imaging, one fluorescent channel (488/564 nm) is selected to detect the green fluorescent signals of rhodamine 110 molecules or FITC probes, and the other fluorescent channel (405/456 nm) is for the blue fluorescence of 4′,6-diamidino-2-phenylindole (DAPI) probes to localize subcellular nuclei. In Figure 2, compared with the control group, confocal imaging presents obvious green fluorescence inside RAW 264.7 cells treated with R-BSA including 50 μg/mL + 24 h, 100 μg/mL + 24 h, or 200 μg/mL + 48 h. However, in Figure S3, no green fluorescent signals originated from FITC probes can be seen in F-BSA groups under the same conditions. To further quantitatively analyze the internalization of BSA or R-BSA by RAW 264.7 cells, the mean fluorescent intensity (MFI) was statistically calculated from ∼100 cells. In Figure 1b, the amount of intracellular R-BSA is raised by 5- to 20-fold upon naive BSA (***P < 0.001). This result demonstrates that the conjugation of lipophilic and cationic rhodamine 110 molecules can efficiently enhance the cellular uptake of BSA by macrophages, due to the high affinity toward phospholipid bilayer membranes with negative charges.^37,38^ Considering the poor internalization of naive BSA by macrophages, we suggest that their cytotoxicity is mainly caused by the extracellular disturbance.^30^ In contrast, it is also plausible to hypothesize that the cytotoxicity of R-BSA is mainly originating from their interference on intracellular metabolism of macrophages. Overall, R-BSA shows better cytocompatibility and higher cellular uptake by macrophages, compared with naive BSA, as illustrated in Scheme 1c,d.

Finally, from the bright-field channel imaging of Figure 2, the polarization of RAW 264.7 cells in R-BSA groups occurs, accompanied with the stretching change of cellular morphology (red arrows), in comparison to unpolarized sphere ones (green arrows). However, in Figure S3, most of the RAW 264.7 cells in control or BSA groups still keep spherical shapes (green arrows), indicating negligible polarization. To further evaluate the polarized degree of macrophages, the mRNA expression of typical M1 polarization associated markers including inducible nitric oxide synthase (iNOS), TNF-α, cluster of differentiation (CD) 86, and IL-23, and the typical M2 polarization associated markers of CD206 was detected by quantitative real-time polymerase chain reaction (q-PCR). Considering a high proportion of living cells (>80%), R-BSA at the concentration of 50 or 100 μg/mL was chosen to be incubated with 5 × 10^5^ per mL RAW 264.7 cells for 24 h. Meanwhile, RAW 264.7 cells stimulated with IFN-γ and LPS were used as a positive control for M1 polarization, ones stimulated with IL-4 were used as a positive control for M2 polarization, and untreated ones were used as a control. In Figure 1c, compared with the control, only the up-regulation of iNOS gene expression as M1 marker and the down-regulation of CD206 gene expression as M2 marker happen in naive BSA groups, with the significant difference of ***P < 0.001 (green asterisks). Similar phenomena have been reported that naive BSA can induce iNOS expression to produce NO in RAW267.4 cells.^39^ However, the gene expression of all M1 biomarkers increases in R-BSA group, accompanied by the down-regulation of M2 biomarker gene expression (***P < 0.001, red asterisks). Especially, compared with naive BSA, R-BSA exhibits stronger regulatory effect on macrophage M1 polarization (***P < 0.001, blue asterisks) at the concentration of 50 or 100 μg/mL. For instance, iNOS mRNA level of R-BSA treatment is elevated by 3- to 10-fold, and CD86 or IL-23 mRNA level also increases by 2- to 5-fold upon BSA treatment. Generally, ROS generation is correlated with the activation and functions of M1 macrophages for antiviral, antimicrobial, and antitumoral activities.^40,41^ Here, intracellular ROS production of RAW 264.7 cells (1 × 10^4^ per mL) treated with R-BSA or BSA at the concentration of 50 or 100 μg/mL for 24 h or at the concentration of 200 μg/mL for 48 h was also measured by dihydroethidium (DHE) assay with the specific detection of cytosolic superoxide production.^42^ In Figure 1d, naive BSA has negligible effect on ROS generation of RAW 264.7 cells. However, compared with naive BSA, R-BSA can induce more ROS production with 1–3 times increasing (***P < 0.001 or **P < 0.01). Taken together, R-BSA is a preferential candidate to mediate macrophages M1 polarization.

### Loading and Release R-BSA by PSiNPs Nanocarriers

**Figure 3:**
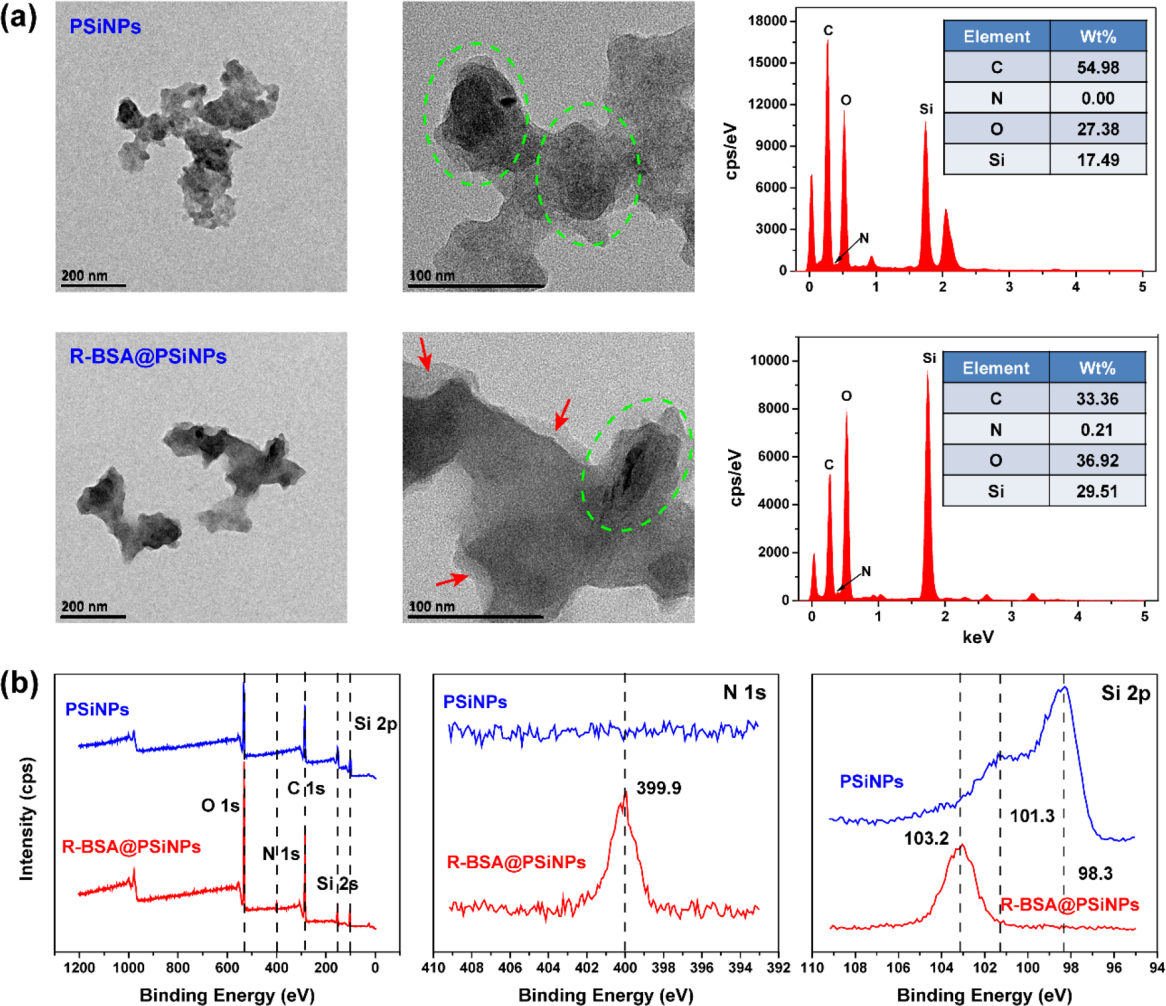
(a) TEM imaging with EDS spectra and (b) full XPS spectra with high-resolution of N 1s and Si 2p of PSiNPs and R-BSA@PSiNPs nanocomposites.

**Figure 4:**
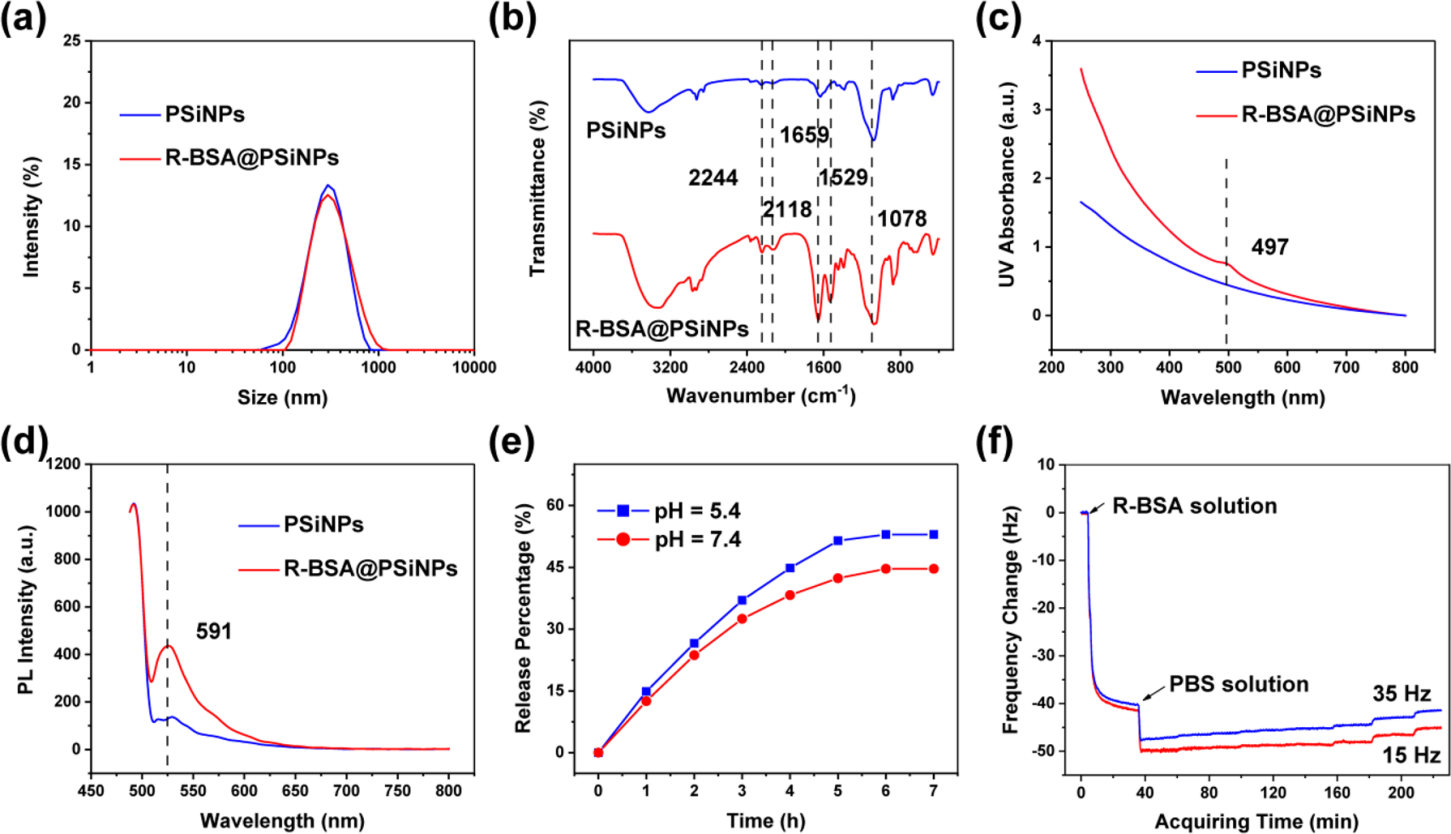
(a) Hydrodynamic size plots, (b) FTIR spectra, (c) UV–vis spectra, and (d) PL spectra excited with 488 nm of PSiNPs and R-BSA@PSiNPs nanocomposites; (e) release profile of R-BSA from R-BSA@PSiNPs nanocomposites under different conditions of pH = 5.4 or 7.4.; and (f) real-time frequency changes of the association and dissociation of R-BSA with PSiNPs.

We ever found that dendritic cells recognized PSiNPs as “foreign body” to efficiently take them up.^22^ Herein, PSiNPs were designed as nanocarriers to deliver R-BSA into macrophages. As illustrated in Scheme 1b, R-BSAs were directly attached onto hydrogen-terminated PSiNPs to fabricate R-BSA@PSiNPs nanocomposites, via hydrophobic interactions reported in our previous studies.^43−45^ Subsequently, TEM and scanning electron microscopy (SEM) with energy dispersive spectrometry (EDS) were utilized to observe the size and morphology and analyze the atomic components of PSiNPs and R-BSA@PSiNPs nanocomposites. In Figure 3a, TEM imaging displays that 50–100 nm silicon nanoparticulate domains marked by green dashed circles are trapped in larger pieces of the porous silicon structure, called as “nanofragments”. In Figure S4, SEM imaging at larger scale also presents similar morphology. Similar PSiNPs with the size larger than 100 nm via this top-down method have been ever reported for *in vivo* imaging.^46^ In Figure 3a, TEM images with higher magnification also display R-BSA layers (marked by red arrows) surrounding PSiNPs cores with the depth of 10–20 nm. And in Figures 3a and S3, their corresponding EDS spectra simultaneously present that the informative signal of rich nitrogen from R-BSA appears in R-BSA@PSiNPs, compared with bare PSiNPs. Next, X-ray photoelectron spectrometry (XPS) was performed to monitor the atomic components of PSiNPs and R-BSA@PSiNPs nanocomposites. In Figure 3b, the survey spectra exhibit the signals of C 1s (284.5 eV), N 1s (399.9 eV), O 1s (533.0 eV), and Si 2p (103.2 eV), consistent with chemical components of R-BSA@PSiNPs nanocomposites. And the corresponding atomic concentrations of these elements were also detected in Table S1. Relative to bare PSiNPs, N 1s signal is obviously found in R-BSA@PSiNPs, with the concentration evolving from 0.00% to 4.12%. And the atomic concentration of silicon correspondingly decreases from 30.46% to 10.05%, due to the limitation of R-BSA encapsulation on the penetration depth of X-ray to detect underlying silicon atoms.^43^ And in Figure 3b, the high-resolution Si 2p spectra of freshly prepared PSiNPs samples show a strong peak at 98.3 eV assigned to the signal of oxidative-free Si–Si(C) and a weak shoulder peak at 101.3 eV assigned to the signal of Si–O. However, for R-BSA@PSiNPs, there is only a strong peak at 103.2 eV, indicating the heavy oxidation of PSiNPs cores. Mean hydrodynamic size and ζ-potential of PSiNPs and R-BSA@PSiNPs were also measured by DLS, recorded in Figure 4a and Table S1. The size of oxidized PSiNPs is 266.7 ± 2.55 nm, and the size of R-BSA@PSiNPs slightly increases to 292.2 ± 2.43 nm. And they have similar PDI values of 0.15 ± 0.01 and 0.15 ± 0.02, indicating good aqueous dispersibility. However, compared with oxidized PSiNPs, the ζ-potential of R-BSA@PSiNPs apparently changes from −29.1 ± 0.60 mV to −2.04 ± 0.12 mV, attributed to the surface coating of R-BSA layers with the ζ-potential of −8.09 ± 0.32 mV.

Moreover, PSiNPs and R-BSA@PSiNPs nanocomposites were also characterized by Fourier transform infrared transmission (FTIR), UV–vis, or PL spectra. In Figure 4b, FTIR spectra of R-BSA@PSiNPs display 1659 and 1529 cm^–1^ assigned to amide I and II bands of R-BSA and 2244, 2118, 1078 cm^–1^ identified as O_3_Si–H, O_2_Si–H_2_, and Si–O–Si stretching from PSiNPs. From UV–vis and PL spectra in Figure 4c,d, the appearance of the absorbance peak at 497 nm and the emission peak at 591 nm in R-BSA@PSiNPs further validate the encapsulation of R-BSA onto PSiNPs. According to the standard curve between the concentration of R-BSA and their PL intensities at 591 nm in Figure S5, the loading efficiency of R-BSA by PSiNPs nanocarriers was calculated as 11.4 ± 2.1% (w/w). And the release profile of R-BSA from PSiNPs nanocarriers was also measured in physiological environments at pH 7.4 or intracellular endo/lysosome environments at pH 5.4. As seen in Figure 4e, in the case of pH 7.4, the maximum release percentage of R-BSA from PSiNPs cores reaches 44.9% after 6 h, with a slight increasing to 53.2% at pH 5.4.

Finally, quartz crystal microbalance (QCM) was performed to *in situ* and real-time track the adsorption and desorption kinetics of R-BSA with PSiNPs, recorded in Figure 4f. QCM can sensitively detect the changes in mass (Δ*m*) on the quartz surfaces via the changes in frequency (Δ*f*) of the oscillating crystal according to Sauerbrey relationship (Δ*m* = −*C*·Δ*f*, where *C* is mass sensitivity constant).^47−50^ According to previous studies,^51^ PSiNPs were first immobilized onto SiO_2_ chips with a preadsorbed polyethyleneimine (PEI) layer via electrostatic interactions. When SiO_2_ chips oscillate at the frequency of 15 or 35 Hz, similar profiles of frequency changes are observed, indicating that these signal changes are attributed to the association and dissociation of R-BSA molecules in media with PSiNPs films on SiO_2_ chips. Taking 15 Hz as an example, in the beginning, the baselines appear to be close to zero after 5 min rinsing with PBS. Subsequently, PBS solution containing 100 μg/mL R-BSA was continuously introduced into cells for 31 min. An initial rapid frequency decreasing to −34.8 Hz happens, followed by a slower frequency decrease to −41.5 Hz as the system saturated. These results demonstrate that R-BSA molecules in media can be efficiently adsorbed onto PSiNPs films and gradually reach dynamic equilibrium. Finally, PBS was again introduced into cells to flush R-BSA molecules detached from PSiNPs films. During this process, the frequency first sharply drops to −49.9 Hz, due to the changes of media from R-BSA solution to PBS.^51^ And then the frequency gradually increases to −45.2 Hz during 189 min rinsing with PBS, indicating the gradual release of R-BSA molecules from PSiNPs films. In contrast to the fast adsorption via hydrophobic interactions, R-BSA molecules are slowly released from PSiNPs. Overall, these above results support that R-BSA can not only be efficiently loaded into PSiNPs to form R-BSA@PSiNPs nanocomposites but also be further released from PSiNPs cores under normal or weak acidic physiological conditions.

### Boosting the Regulatory Effect of R-BSA by PSiNPs Nanocarriers

**Figure 5:**
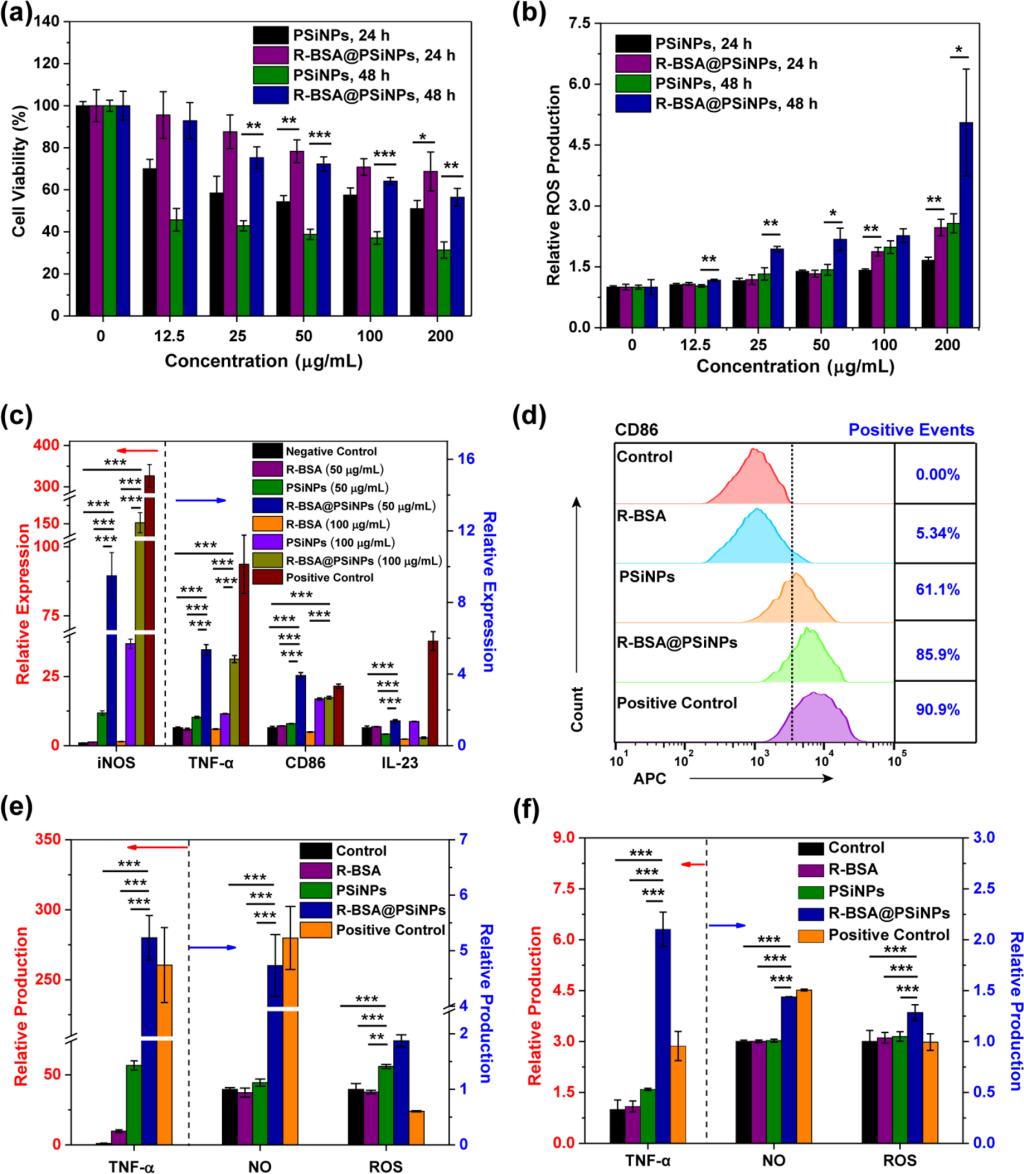
(a) Viability of RAW 264.7 cells treated with PSiNPs or R-BSA@PSiNPs at the concentration range of 0–200 μg/mL for 24 or 48 h (*n* = 6 biological independent samples); (b) ROS production of RAW 264.7 cells with PSiNPs or R-BSA@PSiNPs at the concentration range of 0–200 μg/mL for 24 h (*n* = 4 biological independent samples); (c) relative gene expression of iNOS, TNF-α, CD86, and IL-23 in RAW 264.7 cells treated with 50 or 100 μg/mL R-BSA@PSiNPs, 50 or 100 μg/mL PSiNPs, or R-BSA at the equivalent concentration for 24 h, untreated RAW 264.7 cells as a control, and ones stimulated by LPS + IFN-γ as a positive control (*n* = 3 biological independent samples); and (d) the positive events of CD 86 expression by flow cytometry in RAW 264.7 cells treated with 100 μg/mL R-BSA@PSiNPs, 100 μg/mL PSiNPs, or R-BSA at the equivalent concentration for 24 h, untreated RAW 264.7 cells as a control, and ones stimulated by LPS + IFN-γ as a positive control; (e) production of TNF-α, NO, and ROS in RAW 264.7 cells treated with 100 μg/mL R-BSA@PSiNPs, 100 μg/mL PSiNPs, or R-BSA at the equivalent concentration for 24 h, untreated RAW 264.7 cells as a control, and ones stimulated by LPS + IFN-γ as a positive control (*n* = 4 biological independent samples); and (f) the production of TNF-α, NO, and ROS in peritoneal macrophages harvested from mice with the intraperitoneal injection of 200 μL of R-BSA@PSiNPs (20 mg/kg), 200 μL of PSiNPs (20 mg/kg), R-BSA and at the equivalent concentration, 200 μL of PBS as a control, and 200 μL of LPS (0.5 mg/kg) as a positive control (*n* = 5 biological independent samples). Error bars are based on standard errors of the mean (***P < 0.001, **P < 0.01, or *P < 0.05 by ANOVA with Tukey’s post-test).

First, PSiNPs and R-BSA@PSiNPs at the concentration range of 0–200 μg/mL were incubated with 5 × 10^3^ per mL RAW 264.7 cells for 24 or 48 h to evaluate their cytotoxicity by MTT assay. In Figure 5a, bare PSiNPs have obvious cytotoxicity on RAW 264.7 cells with 48 h treatment, even at the low concentration of 12.5 μg/mL. However, R-BSA@PSiNPs show much better biocompatibility on RAW 264.7 cells. For example, the viability of RAW 264.7 cells treated with 50 μg/mL PSiNPs for 48 h is only 45.7 ± 5.4%. In contrast, the cell viability in R-BSA@PSiNPs group can reach 92.6 ± 8.6% with the significant differences of ***P < 0.001. At higher concentration of 100 or 200 μg/mL for 48 h, the cell viability in R-BSA@PSiNPs group still reaches 64.1 ± 1.7% or 56.5 ± 4.2%, higher than 37.1 ± 3.0% (***P < 0.001) or 31.3 ± 3.9% (**P < 0.01) of PSiNPs group. Even for a 24 h incubation, the cell viability in R-BSA@PSiNPs group is still higher than bare PSiNPs group (**P < 0.01 or *P < 0.05). Our previous studies showed that the cytotoxicity of PSiNPs mainly relied on the strong disruption of their hydrophobic surfaces on cell membranes of macrophages.^28^ Accordingly, it is reasonable to speculate that the surface coatings of R-BSA can efficiently improve the biocompatibility of PSiNPs cores, with preventiion of the disruption on cell membranes. In Figure 5b, ROS production of RAW 264.7 cells (1 × 10^4^ per mL) incubated with PSiNPs and R-BSA@PSiNPs at the concentration range of 0–200 μg/mL for 24 or 48 h was also evaluated by DHE assay, respectively. The results indicate that bare PSiNPs can generate ROS inside RAW 264.7 cells, dependent on the concentration and incubation time. This is because PSiNPs with silicon-based nanostructures can react with oxygen in cell culture medium to produce ROS via quantum confine effect.^52,53^ In contrast, ROS production induced by R-BSA@PSiNPs significantly increases 1- to 3-fold upon bare PSiNPs treatments (**P < 0.01 or *P < 0.05), which is also dependent on the concentration and incubation time. Considering that R-BSA coatings can inhibit the contact of oxygen molecules in media with PSiNPs cores, we speculate that higher ROS production of RAW 264.7 cells treated with R-BSA@PSiNPs is mainly attributed to the attached R-BSA molecules, not PSiNPs cores. Additionally, in Figure 5e, the production of ROS in RAW 264.7 cells treated with 100 μg/mL R-BSA@PSiNPs, 100 μg/mL PSiNPs, or R-BSA at the equivalent concentration was also measured. The highest ROS production in R-BSA@PSiNPs group (***P < 0.001) demonstrates that compared with poor internalization of free R-BSA, PSiNPs nanocarriers can deliver them into RAW 264.7 cells with higher efficiency, leading to more ROS production.

To investigate the regulatory effect of R-BSA@PSiNPs on macrophage M1 polarization, the concentration of 50 or 100 μg/mL and incubation time of 24 h were selected in the following experiments, due to a high viable proportion (>70%) of RAW 264.7 cells. The mRNA expression of iNOS, TNF-α, CD86, and IL-23 in RAW 264.7 cells was evaluated by q-PCR measurements. Meanwhile, RAW 264.7 cells stimulated with IFN-γ and LPS were as a positive control, and untreated ones were as a control. In Figure 5c, compared with the control, iNOS mRNA level of RAW 264.7 cells treated with PSiNPs is elevated by 11- to 37-fold, and TNF-α mRNA level simultaneously increases by 1- to 2-fold. In Figure 5d, flow cytometry analysis also presents that the positive event of CD86 marker in PSiNPs group rises to 61.1%, much higher than 0.0% of the control group. And in Figure 5e, PSiNPs treatments correspondingly induce more than 56-fold increase in TNF-α secretion, relative to the control group. These results show that PSiNPs have an intrinsic immunomodulatory effect to activate innate immune cells, consistent with our previous studies.^21,22,27,28^ In contrast, the gene expression of iNOS, TNF-α, CD86, and IL-23 in R-BSA@PSiNPs groups further increases (***P < 0.001), compared with the control, R-BSA, or PSiNPs groups at the equivalent concentration. For example, iNOS mRNA level is enhanced by 4- to 9-fold higher than bare PSiNPs groups and by 68- to 108-fold higher than free R-BSA groups. TNF-α mRNA expression increases by 2- to 5-fold higher than bare PSiNPs groups or by 5- to 6-fold higher than bare R-BSA groups. CD86 mRNA level also increases by 1- to 4-fold than PSiNPs groups or by 3- to 5-fold than R-BSA groups. In Figure 5d, the positive event of CD86 marker in R-BSA@PSiNPs group can reach 85.9%, higher than 5.34% of R-BSA group or 61.1% of PSiNPs group. And in Figure 5e, the production of NO and TNF-α in R-BSA@PSiNPs groups is also higher than the others groups (***P < 0.001), in agreement with above gene expression analysis. NO production increases by 4- to 5-fold upon PSiNPs group and 5- to 6-fold upon R-BSA group. And they can also secret more TNF-α, with ∼5-fold increasing upon PSiNPs group or ∼29-fold increasing upon R-BSA group. These results confirm that R-BSA@PSiNPs nanocomposites have an enhanced regulatory effect on macrophage M1 polarization, relative to bare PSiNPs or free R-BSA. On this basis, the regulatory effect of R-BSA@PSiNPs nanocomposites *in vivo* was also evaluated in our experiments. The peritoneal cavity was an ideal experimental site to investigate macrophage activation, as shown in previous studies.^54^ First, 200 μL of 20 mg/kg R-BSA@PSiNPs, 20 mg/kg PSiNPs, or R-BSA at the equivalent concentration was administrated into the abdominal of 6-week-old KM mice via the direct intraperitoneal injection, respectively. Here, mice injected with 200 μL of PBS were used as a control, and ones injected with 200 μL of 0.5 mg/kg LPS were as a positive control. After 24 h, all mice were sacrificed to collect macrophages from their peritoneal cavities. The long-term biocompatibility and biodegradability of similar BSA@PSiNPs-based nanosystems via the intravenous injection using the same dose into mice were evaluated in our previous studies.^43,45^ In this experiment, during 24 h postinjection, no abnormal activities including drinking and eating were observed for all mice, indicating the good biosafety of R-BSA@PSiNPs nanocomposites *in vivo*. The production level of NO, ROS, and TNF-α in these murine peritoneal macrophages was subsequently analyzed *ex vivo*. In Figure 5f, the enhanced regulatory effect of R-BSA@PSiNPs on macrophage transition in the abdominal cavities is found again. In contrast to the control, R-BSA, or PSiNPs groups, the secretion of NO and TNF-α in R-BSA@PSiNPs group increases (***P < 0.001), and its ROS production is also the highest (**P < 0.01 or *P < 0.05), in accordance with *in vitro* results. Therefore, we confirm that PSiNPs nanocarriers can boost the regulatory effect of R-BSA on the proinflammatory polarization of macrophages, accompanied by the higher secretion of proinflammatory NO, TNF-α, and ROS.

### Mitochondrial ROS Controlling the Activation of Macrophage Polarization

**Figure 6:**
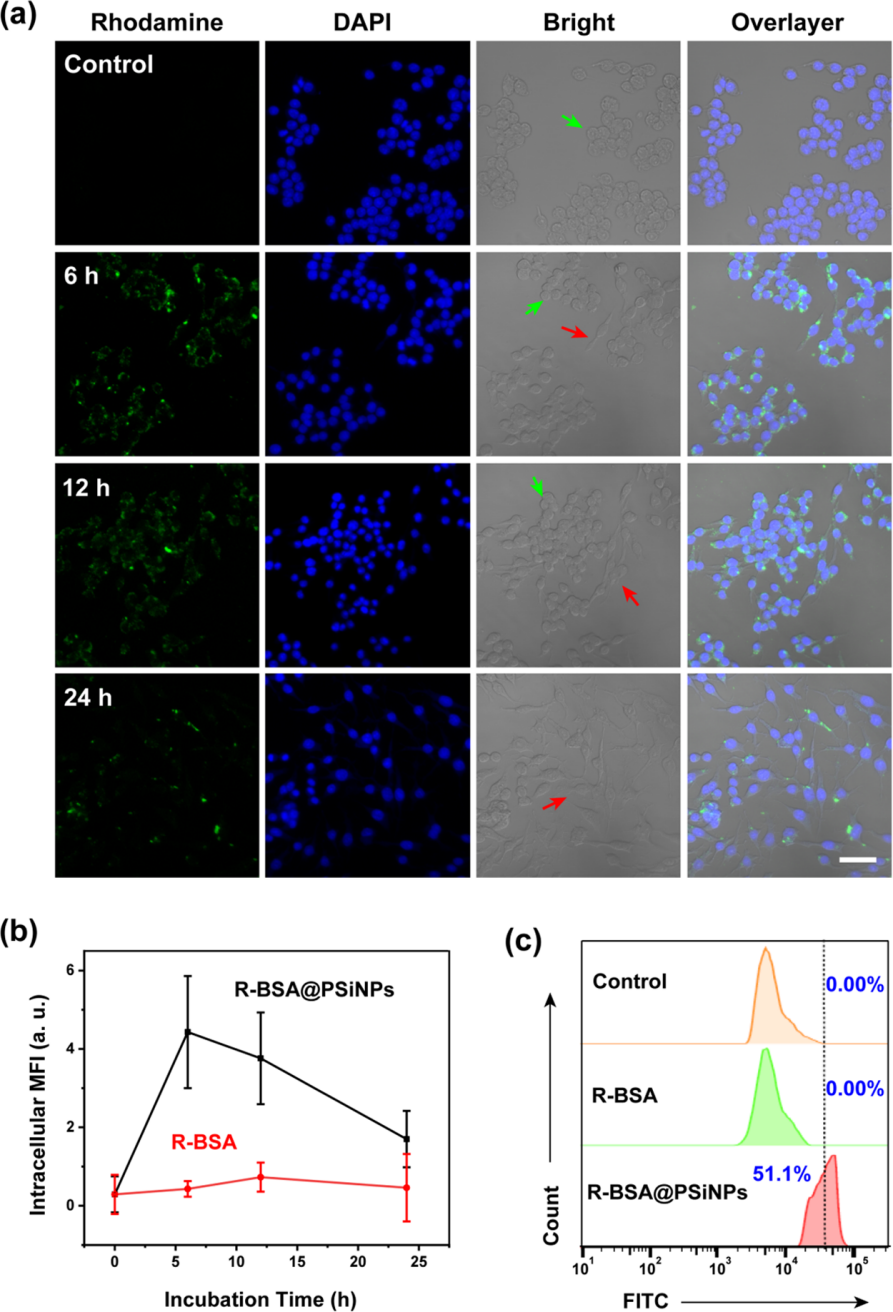
(a) Confocal imaging of RAW 264.7 cells treated with 100 μg/mL R-BSA@PSiNPs for 24 h (scale bar = 40 μm); (b) intracellular MFI from ∼100 cells treated with 100 μg/mL R-BSA@PSiNPs or R-BSA at the equivalent concentration; and (c) flow cytometry analysis of RAW 264.7 cells treated with 100 μg/mL R-BSA@PSiNPs and R-BSA at the equivalent concentration for 24 h.

To shed light on the underlying mechanism of the enhanced immunomodulatory effect of R-BSA@PSiNPs, their interactions with macrophages were first monitored by confocal imaging and flow cytometry. Here, R-BSA@PSiNPs at the concentration of 100 μg/mL or R-BSA at the equivalent concentration were incubated with 2.5 × 10^5^ per mL RAW 264.7 cells for 24 h, respectively. And cell samples were collected for confocal imaging at different time points of 6, 12, and 24 h. Here, one fluorescent channel (488/564 nm) was selected to monitor the green fluorescent signals of rhodamine 110, and the other fluorescent channel (405/456 nm) was for the blue fluorescence of nuclei-targeted DAPI probes. In Figures 6a and S6, confocal imaging observation presents that compared with no green fluorescent signals of R-BSA groups and the control group, strong green fluorescence in R-BSA@PSiNPs groups indicates that they can be efficiently delivered into macrophages by PSiNPs nanocarriers, as seen in Scheme 1d,f. Similar results are also obtained by flow cytometry. In Figure 6c, after a 24 h incubation, in comparison to the control (0.0%) or free R-BSA group (0.0%), FITC-positive (488/520 nm) events of macrophages increase by 51.1%, due to the efficient internalization of R-BSA@PSiNPs. Meanwhile, the endocytosis kinetics was also monitored by intracellular MFI of confocal imaging. As shown in Figure 6b, intracellular MFI in R-BSA group slightly increases within the first 12 h and then gradually decays until 24 h. In contrast, MFI in R-BSA@PSiNPs group quickly reaches the peak within the first 6 h, with ∼10-fold higher than free R-BSA group. Even after 24-h incubation, MFI in R-BSA@PSiNPs group still keeps ∼4-fold stronger upon free R-BSA group. These results show that PSiNPs nanocarriers can accelerate and promote the internalization of R-BSA by macrophages. In addition, the decay of MFI also manifests that macrophage retains the normal digestive activities for exogenous materials.^22^ From the bright field channel of confocal imaging in Figure 6a, the morphological changes of RAW 264.7 cells treated with R-BSA@PSiNPs from spherical shapes (green arrows) to shuttle shapes (red arrows) begin to happen after 6 h, and most of them change their morphology after 24 h. However, no obvious morphological changes appear in free R-BSA groups in Figure S6. This visual evidence can also support the regulatory effect of R-BSA@PSiNPs on macrophage polarization.

**Figure 7:**
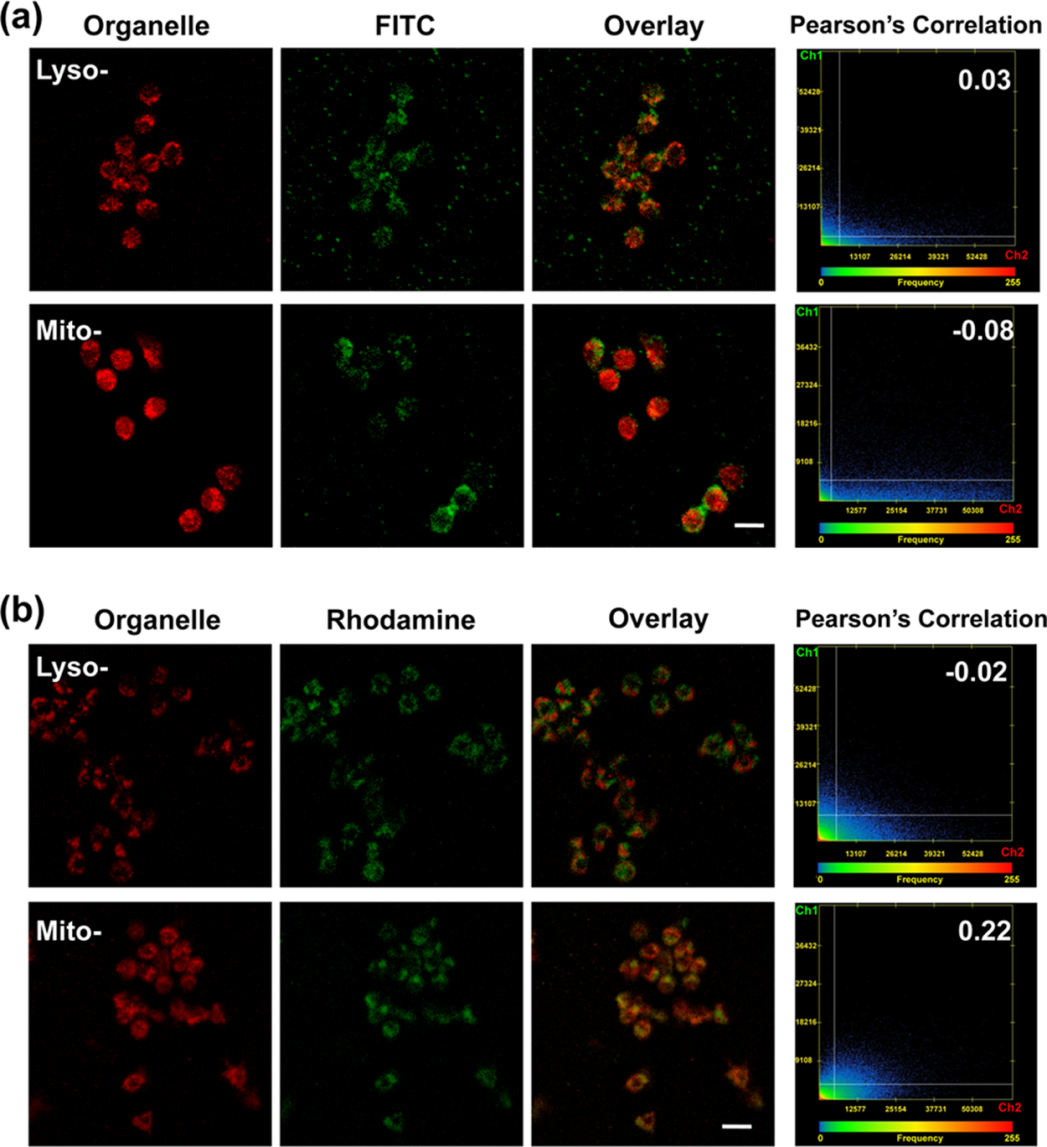
Confocal imaging and the corresponding Pearson’s correlation analysis of RAW 264.7 cells incubated with (a) 100 μg/mL F-BSA@PSiNPs and (b) 100 μg/mL R-BSA@PSiNPs for 12 h after the staining of lyso-/mito-tracker probes, respectively (scale bar = 20 μm).

Considering mitochondrial targeting of R-BSA molecules, the subcellular distribution of R-BSA@PSiNPs nanocomposites inside macrophages was also observed by confocal imaging. In our experiments, 2.5 × 10^5^ per mL RAW 264.7 cells were incubated with R-BSA@PSiNPs at the concentration of 100 μg/mL for 12 h and subsequently stained by LysoTracker or MitoTracker probes for confocal imaging, respectively. And F-BSA@PSiNPs at the same concentration were chosen as a nontargeting control. Green fluorescent channel (488/564 nm) was used to detect the fluorescent signals of intracellular R-BSA or F-BSA, and red fluorescent channel (543/621 nm) was performed for organelle-tracker probes. And Pearson’s correlation between green and red fluorescence was simultaneously calculated to evaluate the organelle colocalization of these internalized nanoparticles. From the merged images in Figure 7a, green fluorescent signals of F-BSA and red fluorescent signals of MitoTracker probes are clearly separated, with Pearson’s coefficient of only −0.08. And the slight overlap between green fluorescence of F-BSA and red fluorescence of LysoTracker probes appears, with Pearson’s coefficient reaching 0.03. In contrast, in Figure 7b, green fluorescent signals of R-BSA obviously overlap red fluorescence of MitoTracker probes with Pearson’s coefficient increasing to 0.22, higher than −0.02 in lysosomes. These results show that intracellular R-BSA@PSiNPs nanocomposites prefer to accumulate toward mitochondria, not lysosomes inside macrophages. As a nontargeting control, F-BSA@PSiNPs appear to locate in the cytosolic compartments of macrophages, not lysosomes or mitochondria, as shown in Scheme 1e,f. The attachment of lipophilic and cationic rhodamine 110 molecules onto nanoparticles can enable them specifically to accumulate in subcellular mitochondria.^37,38^ We adopted R-BSA molecules as templates to synthesize R-BSA@CuS nanocomposites via *in situ* formation of CuS nanoparticles, which show a good mitochondrial-targeting capability with Pearson’s coefficient of 0.68.^35^ Here, we suggest that the large size (292.2 ± 2.43 nm) of R-BSA@@PSiNPs limits their accumulation toward subcellular mitochondria, compared to much smaller R-BSA@CuS nanocomposites (24.7 ± 5.4 nm). On this basis, we hypothesize that only parts of small R-BSA molecules (21.56 ± 0.43 nm) released from PSiNPs cores can accumulate in mitochondria, not large nanoparticles of R-BSA@@PSiNPs.

**Figure 8:**
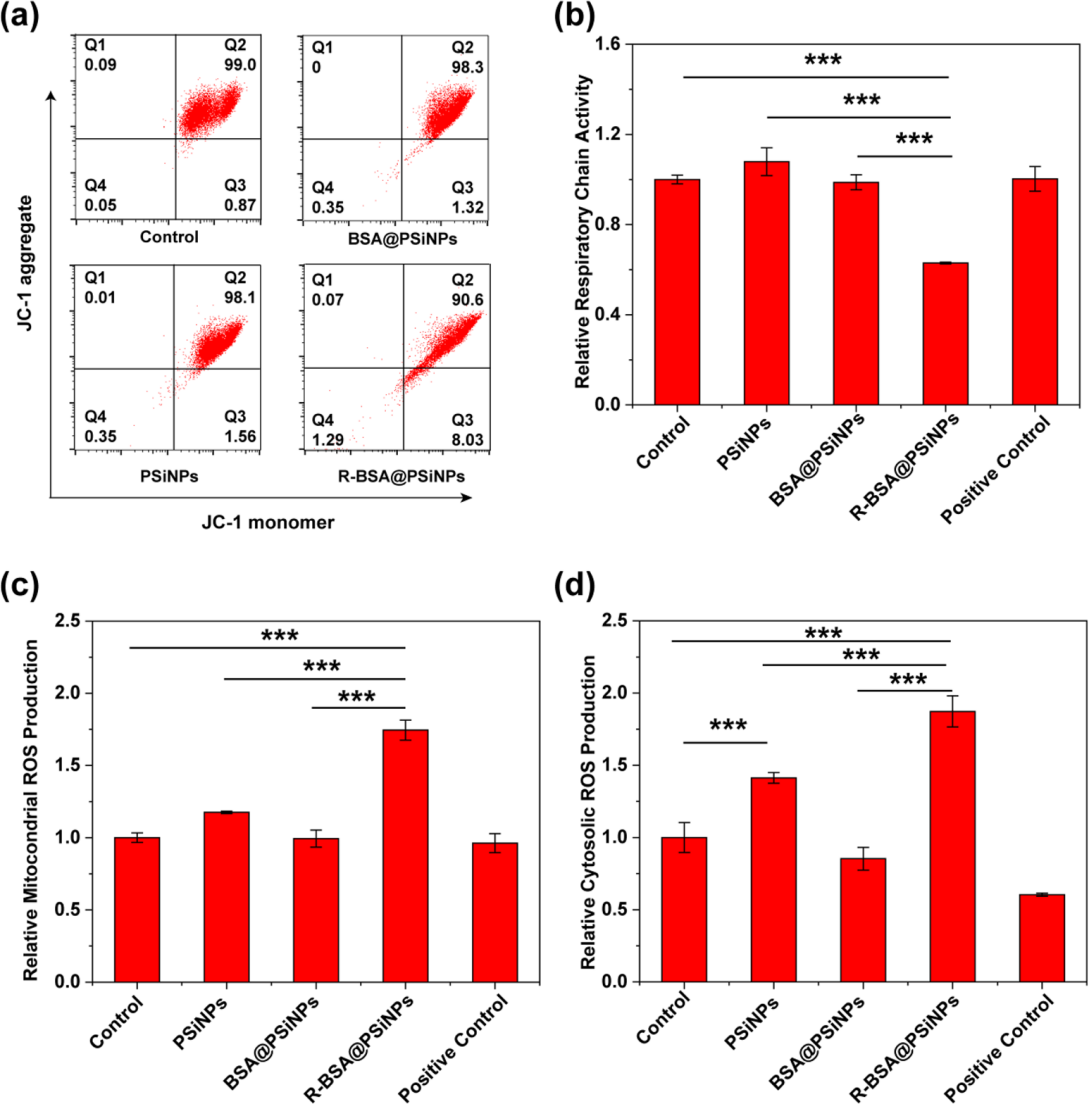
(a) Flow cytometry analysis of RAW 264.7 cells incubated with 100 μg/mL R-BSA@PSiNPs, 100 μg/mL BSA@PSiNPs, or 100 μg/mL PSiNPs for 12 h after the staining of JC-1 probes; (b) respiratory chain activity, (c) mitochondrial ROS, and (d) cytosolic ROS production of RAW 264.7 cells treated with 100 μg/mL R-BSA@PSiNPs, 100 μg/mL BSA@PSiNPs, or 100 μg/mL PSiNPs for 12 h. Untreated RAW 264.7 cells as a control and ones stimulated by LPS + IFN-γ as a positive control (*n* = 4 biological independent samples). Error bars are based on standard errors of the mean (***P < 0.001 or *P < 0.05 by ANOVA with Tukey’s post-test).

Next, the depolarization of mitochondrial membrane potential of macrophages treated with R-BSA@PSiNPs was also monitored in our experiments. Here, 2.5 × 10^5^ per mL RAW 264.7 cells was incubated with R-BSA@PSiNPs at the concentration of 100 μg/mL, BSA@PSiNPs at the concentration of 100 μg/mL, or R-BSA at the equivalent concentration for 12 h, respectively. And these treated cell samples were subsequently stained by 5,5′,6,6′-tetrachloro-1,1′,3,3′-tetraethylbenzimidazolcarbocyanine iodide (JC-1) probes for flow cytometry analysis. JC-1 probes can aggregate in normal mitochondria with polar membrane potential, leading to the emission of strong red fluorescence (525/590 nm). And JC-1 molecules in the monomer state distribute in cytoplasm with strong green fluorescence (488/530 nm), due to the depolarization of mitochondrial membrane potential. In Figure 8a, the cell percentage of untreated macrophages as a control reaches 99.0% in Q2 quadrant, with only 0.87% in Q3 quadrant. In free R-BSA or BSA@PSiNPs groups, there are negligible changes of cell percentage in Q2 (98.1%, 98.3%) or Q3 (1.56%, 1.32%) quadrant. However, for R-BSA@PSiNPs group, the cell percentage in Q2 quadrant decreases to 90.6%, with the increasing to 8.03% in Q2 quadrant. These results show that compared with nontargeting BSA@PSiNPs, mitochondrial-targeting R-BSA@PSiNPs induce the depolarization of mitochondrial membrane potential of macrophages. Without the delivery by PSiNPs nanocarriers, free R-BSA molecules at the equivalent concentration have negligible effect, due to their much lower intracellular concentration. In addition, the respiratory chain activities and cytosolic and mitochondrial ROS production of macrophages treated with R-BSA@PSiNPs were further analyzed, respectively. 1 ×10^4^ per mL RAW 264.7 cells was incubated with R-BSA@PSiNPs (100 μg/mL), BSA@PSiNPs (100 μg/mL), PSiNPs (100 μg/mL), or R-BSA at the equivalent concentration for 12 h, respectively. Meanwhile RAW264.7 cells stimulated with IFN-γ and LPS were used as a positive control, with untreated ones as a control. Here, the respiratory chain activity of these treated cell samples was measured by 7-hydroxy-3*H*-phenoxazin-3-one-10-oxide sodium salt (resazurin) probes (Figure 8b), their mitochondrial superoxide production was measured by MitoSOX red probes (Figure 8c), and cytosolic superoxide production was also measured by DHE probes (Figure 8d).^42^ These results demonstrate that mitochondrial ROS production in R-BSA@PSiNPs group is the highest among these five groups (***P < 0.001), accompanied with the lowest activity of the respiratory chain (***P < 0.001). However, compared with the control, PSiNPs or BSA@PSiNPs groups have no significant effect. For cytosolic ROS generation, compared with the control, cytosolic ROS production in PSiNPs is higher (***P < 0.001). However, R-BSA@PSiNPs group is still the highest among these five groups (***P < 0.001). According to these above results, we can speculate that R-BSA molecules released from PSiNPs cores specifically accumulate in mitochondria, leading to the depolarization of membrane potential and the decreasing of respiratory chain activity. With the dysfunction of the respiratory chain components, the leakage of free electrons can react with molecule oxygen to produce superoxide ion radicals located in mitochondria via the aberrant transfer of these electrons, which can further diffuse to cytosolic compartments inside macrophages.^55^ In contrast, no mitochondrial ROS production occurs in PSiNPs group, while only cytosolic ROS can be generated via their intrinsic quantum confine effect.^56,57^ Combining no positive effect of nontargeting BSA@PSiNPs, these results can confirm that mitochondrial targeting plays a critical role in intracellular ROS generation.

**Figure 9:**
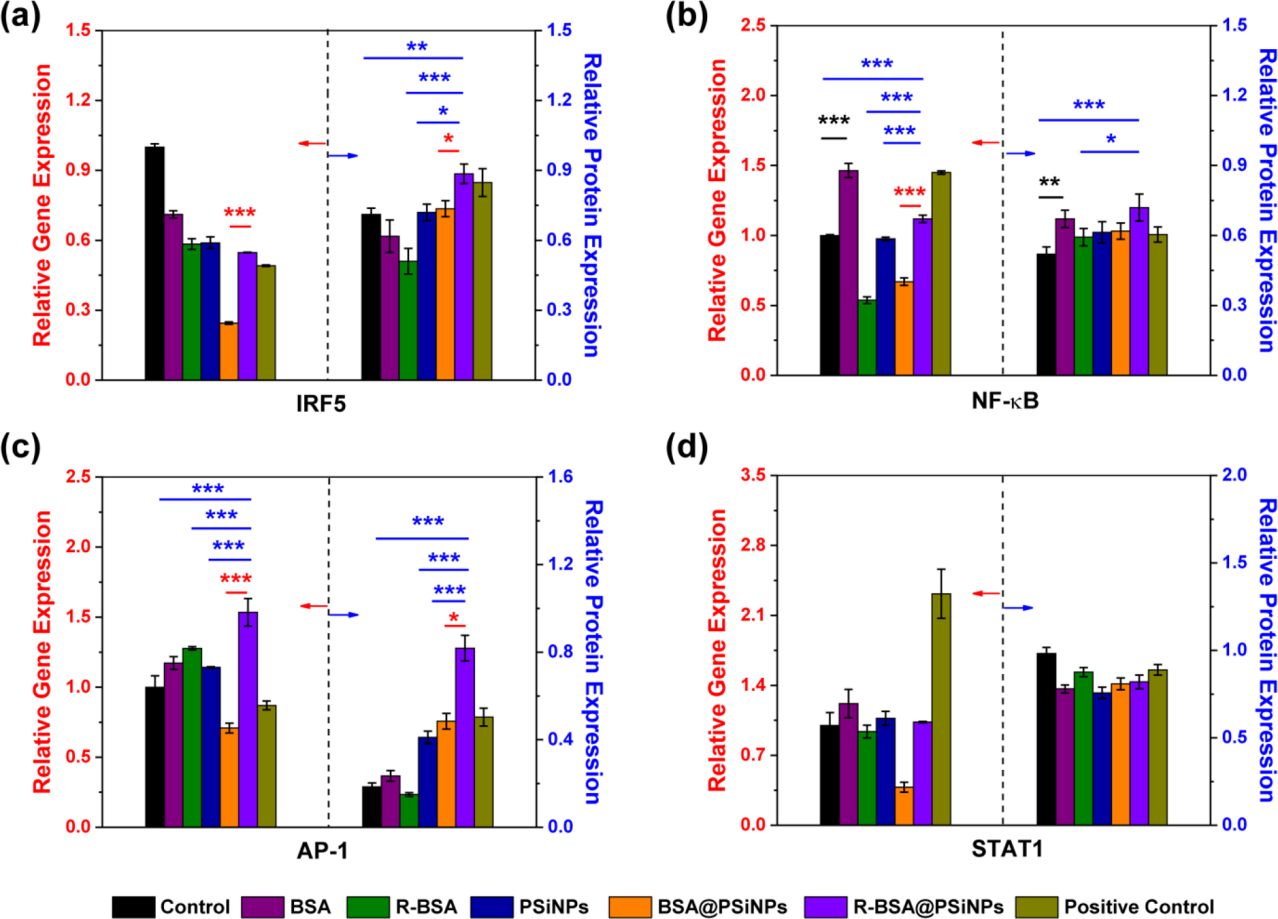
Gene and protein expression levels including (a) IRF5, (b) NF-κB, (c) AP-1, and (d) STAT1 of RAW 264.7 cells incubated with 100 μg/mL R-BSA@PSiNPs, 100 μg/mL BSA@PSiNPs, 100 μg/mL PSiNPs, and BSA or R-BSA at the equivalent concentration for 24 h. Untreated RAW 264.7 cells as a control and ones stimulated by LPS + IFN-γ as a positive control. Error bars are based on standard errors of the mean (***P < 0.001, **P < 0.01, or *P < 0.05 by ANOVA with Tukey’s post-test).

Finally, the activation of four classic signaling transduction pathways including the activation of activator protein 1 (AP-1), interferon regulatory factor 5 (IRF5), NF-κB, and signal transducer and activator of transcription 1 (STAT1) were investigated. First, 5 × 10^5^ per mL RAW 264.7 cells was incubated with 100 μg/mL R-BSA@PSiNPs, 100 μg/mL BSA@PSiNPs, 100 μg/mL PSiNPs, or BSA and R-BSA at the equivalent concentration for 24 h, respectively. And the mRNA expression level of AP-1, IRF5, NF-κB, and STAT1 of as-treated cell samples was analyzed by q-PCR, and their corresponding protein expression level was evaluated by Western blotting assay. Here, macrophages stimulated with IFN-γ + LPS were used as a positive control, with no treatments as a control. According to the results of gene and protein expression shown in Figures 9 and S7, we can find (1) in Figure 9a–c, relative to the control, that the up-regulation of IRF5, NF-κB, and AP-1 expression in the positive control group is attributed to the stimulation of toll-like receptor 4 (TLR4) by LPS and the stimulation of IFN-γ receptor by IFN-γ.^58^ Although the gene expression of STAT1 is upregulated, the protein expression is unexpectedly down-regulated. Therefore, we only confirm that IFN-γ + LPS efficiently activates three pathways of IRF5, NF-κB, and AP-1 in current studies. (2) In Figure 9b, naive BSA can induce the obvious overexpression of NF-κB at gene level (***P < 0.001) or protein level (**P < 0.01) (black asterisk), compared with the control group. This is because BSA molecules can stimulate the receptors located on the membranes of macrophages to active the expression of NF-κB.^30,31,39^ (3) In Figure 9a–c, the expression of IRF5, NF-κB, and AP-1 in R-BSA@PSiNPs group is the highest at gene or protein level, compared with free R-BSA, bare PSiNPs, or the control group (*P < 0.05, **P < 0.01, or ***P < 0.001, blue asterisk). R-BSA@PSiNPs also have negligible regulatory effect to activate the expression of STAT1, similar to IFN-γ + LPS. (4) Compared with BSA@PSiNPs group without mitochondrial targeting, the obvious up-regulated expression of IRF5, NF-κB, and AP-1 at gene or protein level appears in R-BSA@PSiNPs group (*P < 0.05 or ***P < 0.001, red asterisk). This result further confirms that mitochondrial ROS generation in macrophages can activate their M1 polarization, via the downstream signaling cascades of NF-κB, AP-1, and IRF5.^59,60^ And some previous studies also demonstrated that the oxidation of Janus kinase (JAK) 1 and 2, extracellular signal-regulated kinase (ERK) 1/2, or p38 mitogen-activated protein kinases (MAPKs) induced by cytosolic ROS could control the switch M1/M2 transition of macrophages.^61,62^ To further verify these pathways, the inhibition experiments via scavenging ROS with vitamin C (VC) were also carried out. In Figure S8, VC molecules as electron donors can efficiently eliminate intracellular ROS concentrations. For instance, at the concentration of 1.0 μg/mL, VC eliminate 26.4% ROS induced with R-BSA@PSiNPs (***P < 0.001). When the concentration of VC increases to 2.5 or 5.0 μg/mL, they can scavenge 64.0% or 65.0% ROS, with no significant differences. Accordingly, 2.5 μg/mL VC was chosen for the next inhibition experiments. In Figure S9a, ROS scavenging by VC can result in the sharp decreasing of the mRNA level of IRF5, NF-κB, and AP-1 (***P < 0.001). In Figure S9b, ROS scavenging can also inhibit the gene expression of iNOS, IL-23, and TNF-α. In addition, Figure S9c further shows that the gene expression of downstream iNOS, IL-23, and TNF-α also decreases (***P < 0.001), with the adding of the antagonists of IRF5, NF-κB, and AP-1. Altogether, these results fully validate the signal pathway shown in Scheme 1f; that is, R-BSA@PSiNPs can efficiently induce ROS generation via mitochondrial dysfunction, activate the expression of NF-κB, IRF5, and AP-1, and trigger proinflammatory transition of macrophages with cytokines secretion.

### Study Limitations

In our previous studies, we have found that nontargeting BSA@PSiNPs nanosystems with good biosafety *in vivo* had potential applications as “stealth nanocarriers” for drug delivery against cancers.^43^ However, mitochondria-targeted BSA@PSiNPs nanosystem is further developed here to regulate macrophage transition via the interference of mitochondria functions. This strategy is beneficial to design and construct more immunomodulatory nanosystems, beyond the traditional ways of LPS and IFN-γ stimulus on membrane receptors, or cytosolic ROS generation based on catalytic nanoparticles.^56−58,60^ In addition, compared with the poor biocompatibility and loading efficiency of the catalytic nanoparticles or lipopolysaccharide stimulators, this nanosystem composed of PSiNPs and BSA with versatile surface functionalization and good loading capability is an alternative asset for combined immunotherapy.^20,29^ Finally, compared with other supercomplex nanosystems, these commercially available and low-cost materials, such as silicon/silica nanomaterials or albumin proteins were used in our experiments to construct this nanosystem via a simple synthetic method, which is very advantageous for future clinical translation.

## Conclusions

In summary, R-BSA has good mitochondria-targeting capability to generate mitochondrial ROS, which can be further augmented by PSiNPs nanocarriers to efficiently mediate macrophage proinflammatory transition *in vitro* or *in vivo*. Meanwhile, R-BSA coatings can also improve the biocompatibility of PSiNPs cores for macrophages. Moreover, the mechanistic investigations demonstrate that the regulatory effect of R-BSA@PSiNPs mainly relies on mitochondrial ROS-triggering the activation of the signaling transduction pathways including IRF5, NF-κB, and AP-1 inside macrophages, and the production of downstream iNOS, IL-23, and TNF-α. Accordingly, we suggest that this R-BSA@PSiNPs hybrid nanosystem developed here is beneficial for the immunotherapy against infectious or tumoral diseases, due to its strong immunomodulatory effect on macrophage proinflammatory polarization.

## Materials and Methods

### Materials

The single side polished p-type silicon wafers (1–10 Ω̇cm resistivity) were purchased from Hefei Kejing Materials Technology Co. Ltd., China. The BSA was obtained from Sinopharm Chemical Reagent Co. Ltd., China. Sulfo-NHS and EDC were bought from Shanghai Macklin Biochemical Co., Ltd., China. Rhodamine 110 chloride was purchased from Sigma-Aldrich Chemicals Reagent Co. Ltd., USA. The primary antibodies used were anti-STAT1-α, anti-NF-κB, anti-C-JUN, anti-IRF5 purchased from Abcam, U.K. Goat anti-rabbit IgG(H&L)-HRP and anti-tubulin-β were purchased from Bioworld, China. Dulbecco’s modified Eagle’s medium (DMEM), MTT, JC-1, DAPI, LysoTracker red probes, and MitoTracker organelle probes were all obtained from KeyGEN Biotechnology Co. Ltd., China. Defactinib (an inhibitor of IRF5), JSH-23 (an inhibitor of NF-κB), and SP600125 (an inhibitor of AP-1) were obtained from MedChemExpress, USA. Deionized (DI) water (≥18 MΩ̇cm resistivity, Millipore) was used in the experiments.

### Preparation of R-BSA@PSiNPs Nanocomposites

A silicon wafer was immersed in 3:1 (v/v) concentrated H_2_SO_4_/30% H_2_O_2_ at 80 °C for 30 min and then washed repeatedly with DI water. Porous silicon samples were prepared by electrochemical etching in 40% HF/EtOH (1:1, v/v) electrolyte solution at 100 mA/cm^2^ for 15 min. Then the freshly prepared porous silicon powders were ultrasonically dispersed in toluene and ball-milled for 10 h to obtain the freshly prepared PSiNPs. About 14.0 mg of rhodamine 110, 8.6 mg of sulfo-NHS, and 7.2 mg of EDC were dissolved in 1.5 mL of dimethylformamide (DMF) solution, which was continuously stirred for 4 h in dark. The resultant mixture was dropwisely added into 4.5 mL of 15.6 mg/mL BSA aqueous solution and then continuously stirred for 12 h in dark. Finally, the reaction solution was repeatedly dialyzed with dialysis membrane (MWCO = 8–14 kDa) to prepare R-BSA nanocomposites, which was freeze-dried and stored at 4 °C for the following experiments. Next, 500 μg of R-BSA or BSA was ultrasonicated in the PSiNPs aqueous solution (1 mg/mL) for 30 min to fabricate the R-BSA@PSiNPs and BSA@PSiNPs nanocomposites. The R-BSA@PSiNPs and BSA@PSiNPs nanocomposites were repeatedly washed and ultrasonicated in the DI water. To prepared F-BSA nanocomposites, 50 mg of BSA was dissolved in 10 mL of carbonate buffer (pH 9.0–9.5) and then dropwise added with 0.25 mL of 1 mg/mL FITC in dimethylsulfoxide (DMSO) solution to be stirred at 4 °C in dark. After 24 h, the reaction solution was repeatedly dialyzed to prepare F-BSA nanocomposites and then freeze-dried and stored at 4 °C for the next experiments. UV–vis adsorption spectra were measured using a spectrophotometer (Lambda 950, PerkinElmer, USA). PL spectra were recorded by a fluorescence spectrometer (LS55, PerkinElmer, USA). The morphology of nanocomposites was observed with a field-emission SEM instrument (Regulus 8100, Hitachi, Japan) and high-resolution TEM instrument (JEM-2100 UHR, JEOL, Japan) with the accelerating voltage of 200 kV. XPS results were recorded with Kratos AXIS Ultra DLD system (Shimadzu, Japan) with a monochromatic Al Kα X-ray beam (1.5 × 10^3^ eV). Size and ζ-potential of nanoparticles were analyzed with DLS measurements (Zetasizer Nano ZS, Malvern Instruments, U.K.), according to the NIST-NCL joint assay protocol (PCC-1, version 1.2).^22^

### QCM Measurements

The adsorption and desorption of R-BSA were conducted on Q-sense E4 system (Gothenburg, Sweden) with shear flow cells and piezoelectric quartz crystal sensors at 25.0 ± 0.1 °C. At first, the PSiNPs aqueous solution (1 mg/mL) was spin-coated (WS-650SX-6NNP spin coater, Laurell Technologies, North Wales, PA, USA) at 3000 r/min on SiO_2_ chips with a preadsorbed PEI layer. The SiO_2_ chips coated with PSiNPs were mounted in the flow cells, and then the PBS solution was introduced into the cells. In the case of stable baselines, PBS solution with adding 100 μg/mL R-BSA was introduced into the cells. With the appearance of dynamic equilibrium, PBS solution was introduced again to continuously flush SiO_2_ chips. The frequency changes at 15 and 35 MHz were recorded in current studies.

### Cell Viability

RAW 264.7 cells were cultured in DMEM medium which was supplemented with 10% fetal bovine serum and 1% penicillin–streptomycin at 37 °C under a humidified atmosphere with 5% CO_2_. RAW 264.7 cells (5 × 10^3^ cells per mL) were dispersed within 96-well plate to a total volume of 100 μL per well. After 24 h, their culture medium was replaced by the medium containing BSA, R-BSA, PSiNPs, BSA@PSiNPs, and R-BSA@PSiNPs with different concentrations (0–200 μg/mL) for 24 or 48 h. After washing with blank culture medium, 100 μL of culture medium containing 10 μL of MTT solution (5 mg/mL) was then added, followed by incubation for 4 h to allow the formation of formazan dye. After removing the culture medium, 150 μL of DMSO was added to each well to dissolve the formazan crystals. Absorbance was measured at 570 nm in a microplate photometer (Filter Max F5, Molecular Devices, USA). Cell viability values were determined according to the following formula:
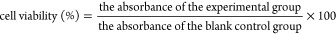


### Gene Expression Analysis

Gene expression of CD206, iNOS, CD86, IL-23, CXCL10, CCL2, TNF-α, and four transcription factors (STAT1, NF-κB, AP-1, and IRF5) was evaluated by qPCR. 5 ×10^5^ per mL RAW 264.7 cells was treated with BSA (9 μg/mL), R-BSA (11 μg/mL), PSiNPs (100 μg/mL), BSA@PSiNPs (100 μg/mL), and R-BSA@PSiNPs (100 μg/mL) for 24 h. Then the cells were harvested followed by total RNA extraction reagent (Vazyme Biotech, China) and the protocol was followed according to the manufacturer’s instructions, and then RNA was quantified with Nanodrop (2000c, Thermo Scientific, USA). 1 μg of total RNA was reverse-transcribed into complementary DNA with reverse transcription kits (Vazyme Biotech, China). qPCR was performed on RT-PCR system (C1000, Bio-Rad, USA,) with use of a qPCR kit (Vazyme Biotech, China) and primers described in Table 1. The comparative *C*_T_ method ( method) was used to analyzed qPCR data.^63^

**Table 1:** The qPCR Primers Used for RT-PCR Analysis in This Study

	primer	
	forward (5′–3′)	reverse (5′–3′)
GAPDH	GCAAATTCAACGGCACAGTCAAG	GGTACAAACACTACCCACACTTG
iNOS	TGCTTTGTGCGAAGTGTCAG	CCCTTTGTGCTGGGAGTCAT
CD86	TGTTTCCGTGGAGACGCAAG	TTGAGCCTTTGTAAATGGGCA
TNF-α	GCCTATGTCTCAGCCTCTTCTC	GCCATTTGGGAACTTCTCATCC
CD206	GCTGGCGAGCATCAAGAGTA	AGGAAACGGGAGAACCATCAC
IL-23	CTGCTCTGTCCCTCAGTTCTAA	TTGTCAGTTCGTATTGGTAGTCC
AP-1	TTGTTACAGAAGCGGGGACG	GAGGGCATCGTCGTAGAAGG
NF-κB	CCTGCTTCTGGAGGGTGATG	GCCGCTATATGCAGAGGTGT
IRF5	CCCTGTCCCAGACCCAAATC	AGGTCCGTCAAAGGCAACAT
STAT1	GTCATCCCGCAGAGAGAACG	GCAGAGCTGAAACGACCTAGA

### Measurement of NO and TNF-α

RAW 264.7 cells were seeded in a 96-well plate at a density of 1 × 10^4^ cells per well. Then, the cells were treated with BSA (9 μg/mL), R-BSA (11 μg/mL), PSiNPs (100 μg/mL), BSA@PSiNPs (100 μg/mL), R-BSA@PSiNPs (100 μg/mL), LPS (100 ng/mL) and IFN-γ (25 ng/mL), or IL-4 (25 ng/mL) for 24 h. Because NO molecules are very unstable in aqueous solution to form nitrite, cell culture supernatants were collected and quantified spectrophotometrically using the Griess reagent (Beyotime Biotech, China) to analyze the NO amount. The absorbance intensity of 540 nm was measured by multifunctional enzyme marker (Infinite 200Pro, Tecan, Swiss). The relative NO production was calculated according to the following formula:
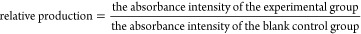


To confirm the production of the TNF-α, the cell culture supernatants were assayed using precoated enzyme linked immunosorbent assay (ELISA) kits (MultiSciences Biotech, China) following the manufacturer’s instructions. The absorbance intensity at 450 and 630 nm was measured by multifunctional enzyme marker (Infinite 200Pro, Tecan, Swiss). And the final absorbance intensity = absorbance intensity at 450 nm – absorbance intensity at 630 nm. Finally, the relative TNF-α production was calculated according to the following formula:
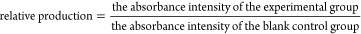


### Cytosolic ROS Measurements

RAW 264.7 cells were seeded in a 96-well plate at a density of 1 × 10^4^ cells per well and incubated at 37 °C for 24 h. Then, the cells were washed twice with PBS followed by incubation with fresh medium containing PSiNPs, R-BSA@PSiNPs with different concentrations (0–200 μg/mL) for 24 or 48 h. After a certain period, the medium was removed and 20 μmol/L of DHE (KeyGen Biotech, China) in fresh medium was added to cells for 20 min of incubation at 37 °C. Afterward, the DHE solution was removed and washed twice with fresh medium. The intensity of fluorescence at 605 nm with excitation peak of 518 nm was measured by multifunctional enzyme marker (Cytation 3, Biotek, USA). And the relative ROS production is according to the following formula:
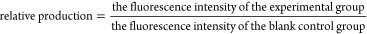


### Mitochondrial ROS Measurements

RAW264.7 cells were seeded in a 96-well plate at a density of 1 × 10^4^ cells per well. Then, the cells were treated with R-BSA@PSiNPs (100 μg/mL), PSiNPs (100 μg/mL), BSA@PSiNPs (100 μg/mL), and LPS (100 ng/mL) + IFN-γ (25 ng/mL) for 24 h. And culture medium was removed, and 5 μmol/L of MitoSOX (Invitrogen, USA) in fresh medium was added for 10 min at 37 °C. Subsequently, MitoSOX solution was removed and washed twice with fresh medium. The intensity of fluorescence (510/580 nm) was measured by multifunctional enzyme marker (Cytation 3, Biotek, USA). And the relative production is according to the following formula:
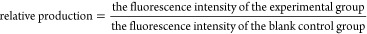


### Respiratory Chain Activity

RAW264.7 cells were seeded in a 96-well plate at a density of 1 × 10^4^ cells per well. Then, the cells were treated with R-BSA@PSiNPs (100 μg/mL), PSiNPs (100 μg/mL), BSA@PSiNPs (100 μg/mL), and LPS (100 ng/mL) + IFN-γ (25 ng/mL) for 24 h. And culture medium was removed, and 6 μmol/L of resazurin (Aladdin, China) in fresh medium was added for 5 min at 37 °C. The intensity of fluorescence (510/595 nm) was measured by multifunctional enzyme marker (Cytation 3, Biotek, USA). And the respiratory chain activity is according to the following formula:
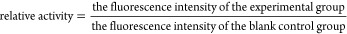


### Confocal Imaging Observation

To observe the subcellular distribution of R-BSA@PSiNPs nanocomposites, cells were plated into 6-well culture plates, and the number of cells per well was 2.5 × 10^5^ cells. After a 12 h incubation, cell culture medium was replaced by the medium containing 100 μg/mL R-BSA@PSiNPs or 100 μg/mL F-BSA@PSiNPs nanocomposites, respectively. After 6 h, these cell samples were washed by PBS and stained by DAPI, MitoTracker orange or LysoTracker red probes for confocal imaging (LSM710 NLO, Zeiss, Germany), respectively.

### Flow Cytometry Tests

2.5 × 10^5^ per mL RAW 264.7 cells was treated with R-BSA (11 μg/mL), PSiNPs (100 μg/mL), R-BSA@PSiNPs (100 μg/mL), LPS (100 ng/mL) + IFN-γ (25 ng/mL) for 24 h. Then the cells were harvested and washed with PBS solution followed by the staining with anti-CD86-APC antibodies (BioLegend, USA) for flow cytometry analysis (Influx, BD Biosciences, USA). To detect the depolarization of mitochondrial membrane potential, 2.5 × 10^5^ per mL RAW 264.7 cells was treated with BSA (9 μg/mL), R-BSA (11 μg/mL), PSiNPs (100 μg/mL), BSA@PSiNPs (100 μg/mL), or R-BSA@PSiNPs (100 μg/mL) for 24 h. Then these cell samples were washed with fresh culture medium and stained with JC-1 probes for flow cytometry analysis (Influx, BD Biosciences, USA).

### Western Blotting Assays

RAW 264.7 cells were plated into 6-well culture plates at a density of 5 × 10^5^ cells per well and incubated at 37 °C. After 24 h, cells were treated with BSA (9 μg/mL), R-BSA (11 μg/mL), PSiNPs (100 μg/mL), BSA@PSiNPs (100 μg/mL), R-BSA@PSiNPs (100 μg/mL), or LPS (100 ng/mL) + IFN-γ (25 ng/mL) for 24 h. Then these cells were washed twice with PBS solution and incubated in radioimmunoprecipitation assay (RIPA) lysis buffer (Beyotime Biotech, China) with 1% phenylmethylsulfonyl fluoride (Beyotime Biotech, China) on ice for 20 min. Bicinchoninic acid (BCA) protein quantification kits (Vazyme Biotech, China) were employed to measure total protein concentration of each sample. About 30 μg protein of cell lysate was prepared for gel electrophoresis. After electrophoresis, the resulting gel was transferred to a polyvinylidene fluoride (PVDF) membrane (Millipore, USA). Then, the PVDF membrane was blocked by 5% nonfat milk for 1 h at room temperature. After washing with Tris-buffered saline with Tween-20 (TBST), the PVDF membrane staining was performed by incubation of primary antibody in 5% nonfat milk at 4 °C overnight and then washed with TBST for three times. The PVDF membrane was further incubated with corresponding secondary antibodies for 1 h at room temperature. After washing with TBST again, the PVDF membrane was added with enhanced chemiluminescence reagents. The blots were detected by using chemiluminescence image analysis system (Tanon 4600, China). The gray scale value of every band was analyzed using ImageJ software. And the quantitative analysis of protein expression was determined using the following formula:
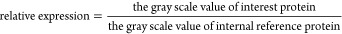


### Inhibition Experiments

5 × 10^5^ per mL RAW 264.7 cells were treated with BSA (9 μg/mL), R-BSA (11 μg/mL), PSiNPs (100 μg/mL), BSA@PSiNPs (100 μg/mL), R-BSA@PSiNPs (100 μg/mL), R-BSA@PSiNPs (100 μg/mL) + VC (2.5 mmol/L), R-BSA@PSiNPs (100 μg/mL) + IRF5 inhibitors (1 μmol/L), R-BSA@PSiNPs (100 μg/mL) + NF-κB inhibitors (20 μmol/L), R-BSA@PSiNPs (100 μg/mL) + AP-1 inhibitors (20 μmol/L) for 24 h. Then the cells were collected follow by total RNA extraction reagent (Vazyme Biotech, China) and the protocol was followed according to the manufacturer’s instructions, and then RNA was quantified with Nanodrop (2000c, Thermo Scientific, USA). 1 μg of total RNA was reverse-transcribed into complementary DNA with reverse transcription kits (Vazyme Biotech, China). qPCR was performed on RT-PCR system (C1000, Bio-Rad, USA,) with use of a qPCR kit (Vazyme Biotech, China) and primers described above. And the comparative *C*_T_ method ( method) was also used to analyze qPCR data.

### Polarization of Peritoneal Macrophages *in Vivo*

To study the polarization of peritoneal macrophages, 25 KM mice with the age of 6 weeks were divided into five groups and intraperitoneally treated with PBS, R-BSA (2 mg/kg), PSiNPs (20 mg/kg), R-BSA@PSiNPs (20 mg/kg), LPS (0.5 mg/kg), respectively. Macrophages were harvested from peritoneal lavage after 24 h postinjection. The cells were washed with DMEM twice and resuspended in DMEM supplemented with 10% FBS and then incubated again at 37 °C for 6 h. Due to the adhering nature of macrophages,^64,65^ the adherent cells were further harvested for the ROS, NO, and TNF-α analysis *ex vivo*, using the above-described method.

### Animal License and Permissions

All animal experiments were reviewed and approved by the Experimental Animal Committee of the KeyGEN Biotechnology Co. Ltd., China. The Animal Ethics Review Approval Number is IACUC-007-7. All the female KM mice with the age of 6 weeks were obtained from SPF (Beijing) Biotechnology Co., Ltd.

### Statistical Analysis

In our studies, all data with mean ± standard deviation were statistically analyzed using SPSS statistics software (19.0 edition), and their statistically significant differences (***p < 0.001, **p < 0.01, *p < 0.05, or NS (nonsignificant difference, *p* > 0.05)) were calculated using the Tukey’s post-test method.

## Supporting Information Available

Additional figures and tables including UV–vis, PL spectra, and DLS results of BSA, R-BSA, and F-BSA samples; TEM imaging of BSA and R-BSA samples; confocal imaging of RAW 264.7 cells treated with F-BSA or R-BSA; SEM with EDS spectra of PSiNPs and R-BSA@PSiNPs nanocomposites; quantitative results of XPS and DLS of PSiNPs and R-BSA@PSiNPs nanocomposites; and qPCR data of ROS scavenging and inhibition experiments (PDF)
