# Supplementary material for: Porous Silicon Nanocarriers Boost the Immunomodulation of Mitochondria-Targeted Bovine Serum Albumins on Macrophage Polarization
Source: ACS Nano. 2023 Jan 4;17(2):1036–53. doi: 10.1021/acsnano.2c07439 (PMC9878978; doi:10.1021/acsnano.2c07439)
Supplement: Supplementary file 1 — nn2c07439_si_001.pdf [file nn2c07439_si_001.pdf]

Supporting Information

Porous Silicon Nanocarriers Boost the Immunomodulation of Mitochondria-Targeted Bovine Serum Albumins on Macrophage Polarization

Jialiang Li,[†] Jiqiang Fan,[§] Yan Gao,[†] Shuodan Huang,[†] Di Huang,[†] Jiachen Li,[#] Xiaoyu Wang,[†] Hélder A. Santos,[*,#] Pingping Shen,[*,‡,§] and Bing Xia[*,†]

[†]College of Science, Nanjing Forestry University, Nanjing 210037, China

[‡]Department of Geriatric Medicine, The Second Affiliated Hospital and Yuying Children's Hospital of Wenzhou Medical University, Wenzhou 325027, China

[§]State Key Laboratory of Pharmaceutical Biotechnology and the Comprehensive Cancer Center, Nanjing Drum Tower Hospital, the Affiliated Hospital of Nanjing University Medical School, Nanjing University, Nanjing 210023, China

[#]Department of Biomedical Engineering, W.J. Kolff Institute for Biomedical Engineering and Materials Science, University Medical Center Groningen/University of Groningen, Ant. Deusinglaan 1, 9713 AV Groningen, The Netherlands

SUPPLEMENTARY FIGURES AND TABLES

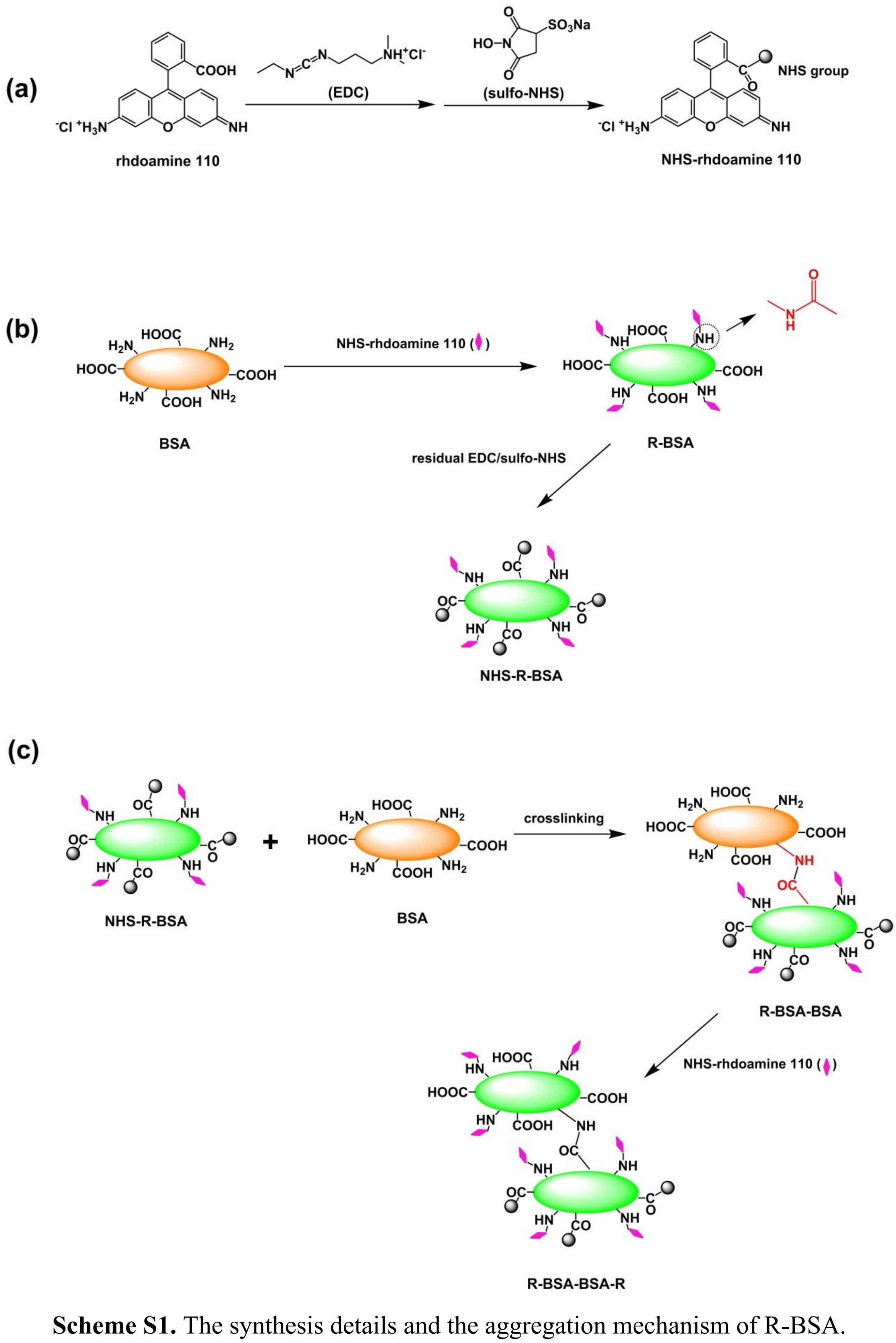

Scheme S1. The synthesis details and the aggregation mechanism of R-BSA.

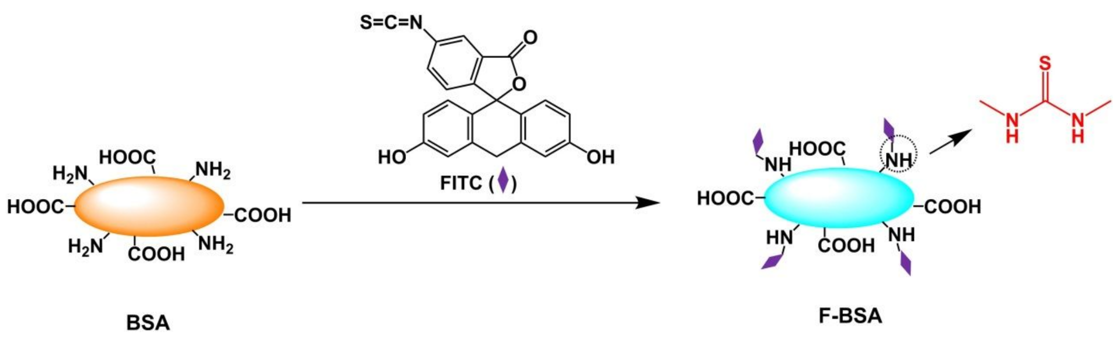

Scheme S2. The synthesis details of F-BSA

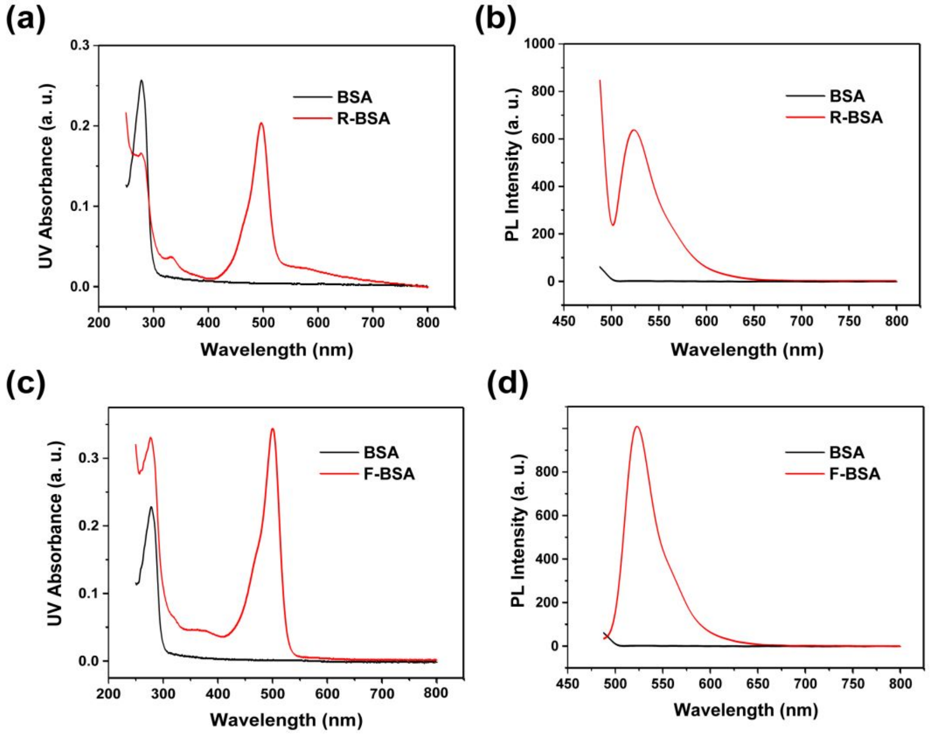

Figure S1. (a) UV-vis and (b) PL spectra (448 nm excitation) of BSA and R-BSA samples; (c) UV-vis and PL spectra (448 nm excitation) of BSA and F-BSA samples.

(a)

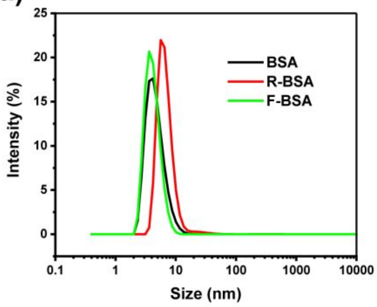

(b)

Materials	Size (nm)	Zeta Potential (mV)	PDI
BSA	6.86 ± 0.47	-9.05 ± 0.55	0.26 ± 0.02
R-BSA	21.56 ± 0.43	-8.09 ± 0.32	0.26 ± 0.02
F-BSA	7.97 ± 0.29	-11.8 ± 1.5	0.28 ± 0.01

(c)

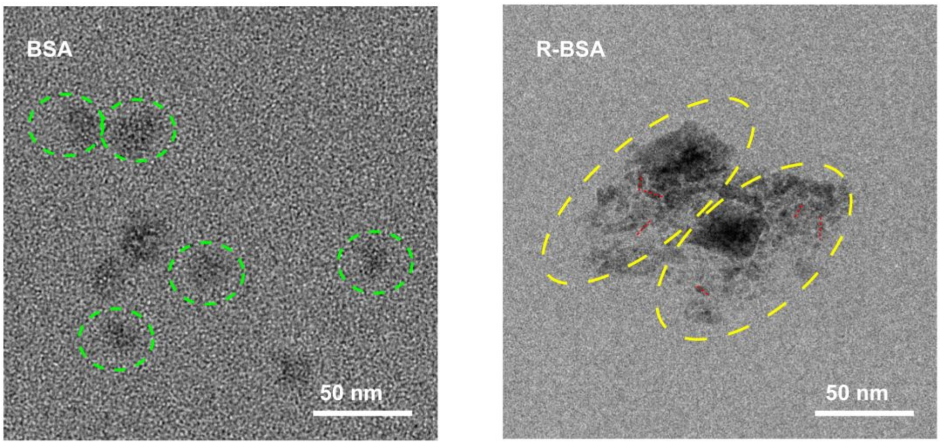

Figure S2. (a) Hydrodynamic size plots of BSA, F-BSA and R-BSA samples; (b) DLS results of BSA, F-BSA and R-BSA samples; and (c) TEM images of BSA and R-BSA samples.

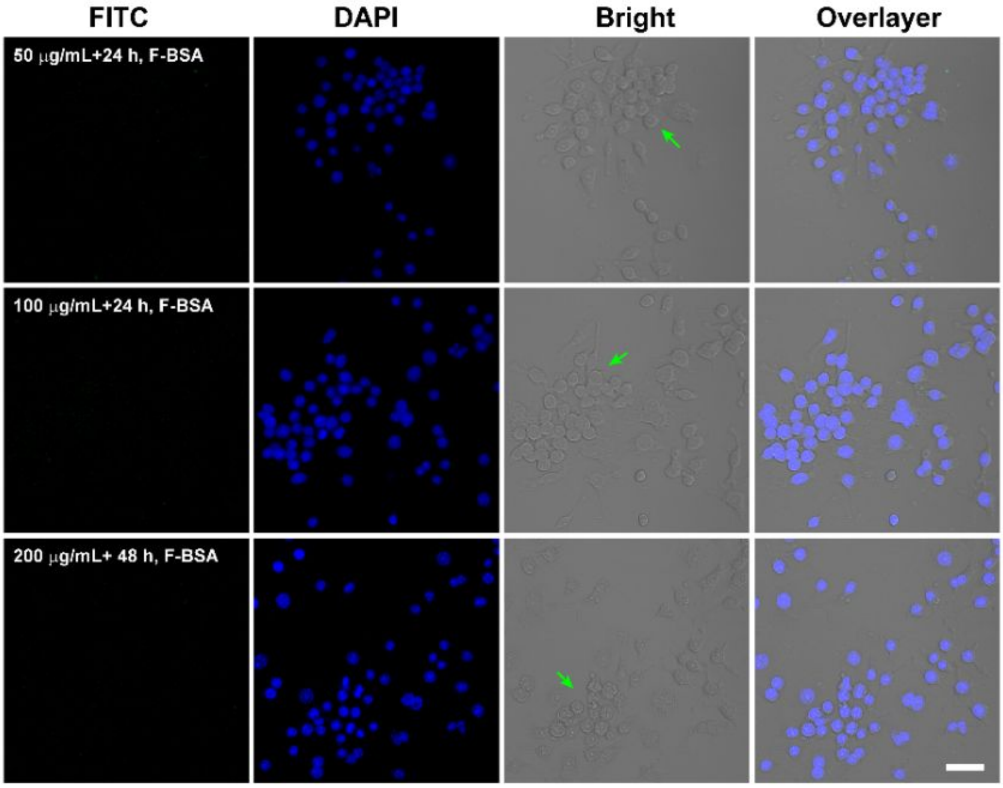

Figure S3. Confocal imaging of the RAW 264.7 cells with F-BSA treatments including 50 μg/mL + 24 h, 100 μg/mL + 24 h, and 200 μg/mL + 48h (scale bar = 40 μm).

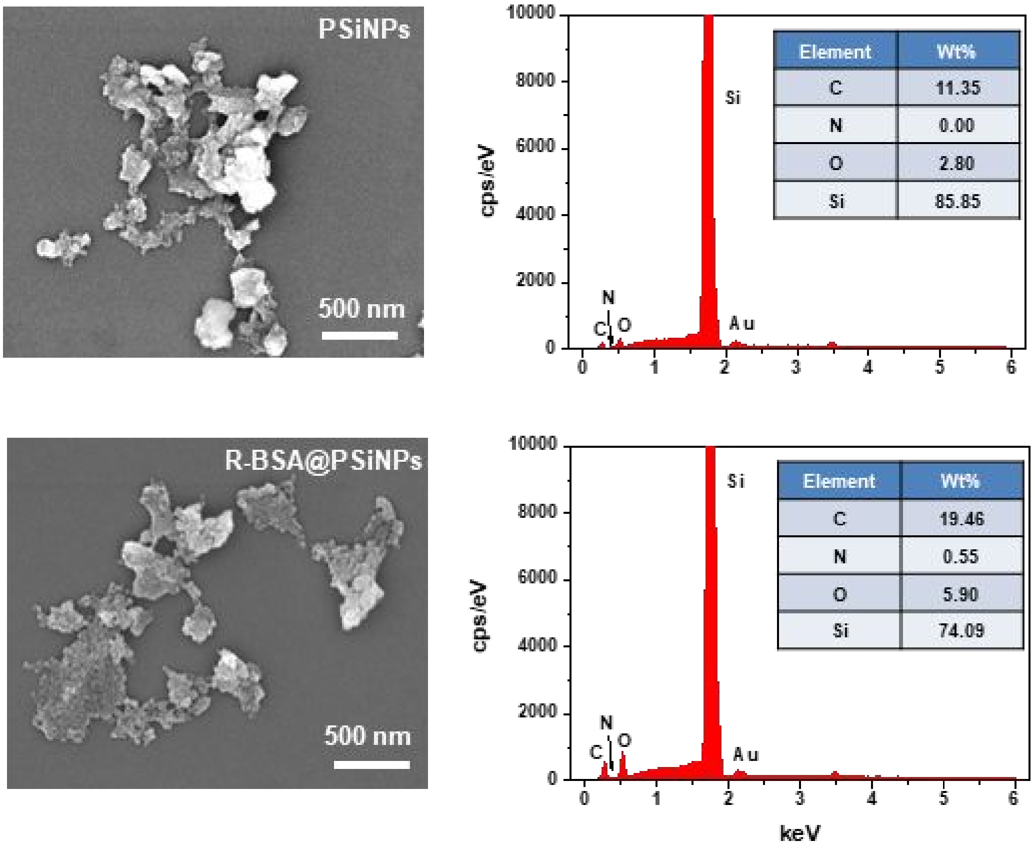

Figure S4. SEM imaging with EDS spectra of PSiNPs and R-BSA@PSiNPs nanocomposites.

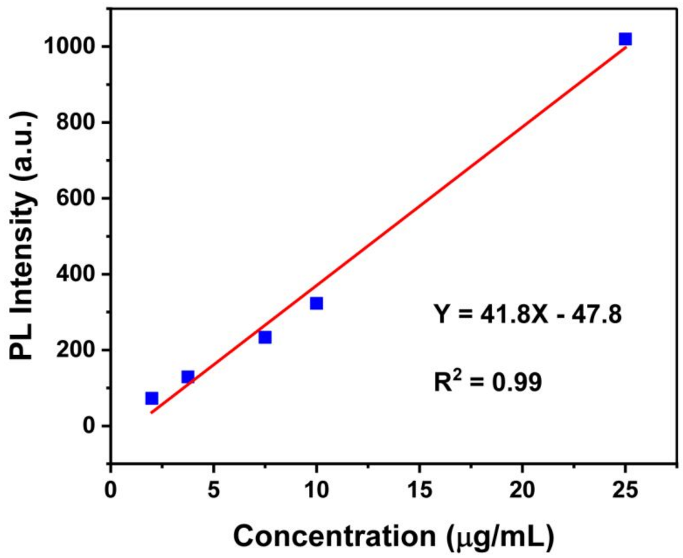

Figure S5. The standard curve plot between PL intensity of R-BSA and their concentration.

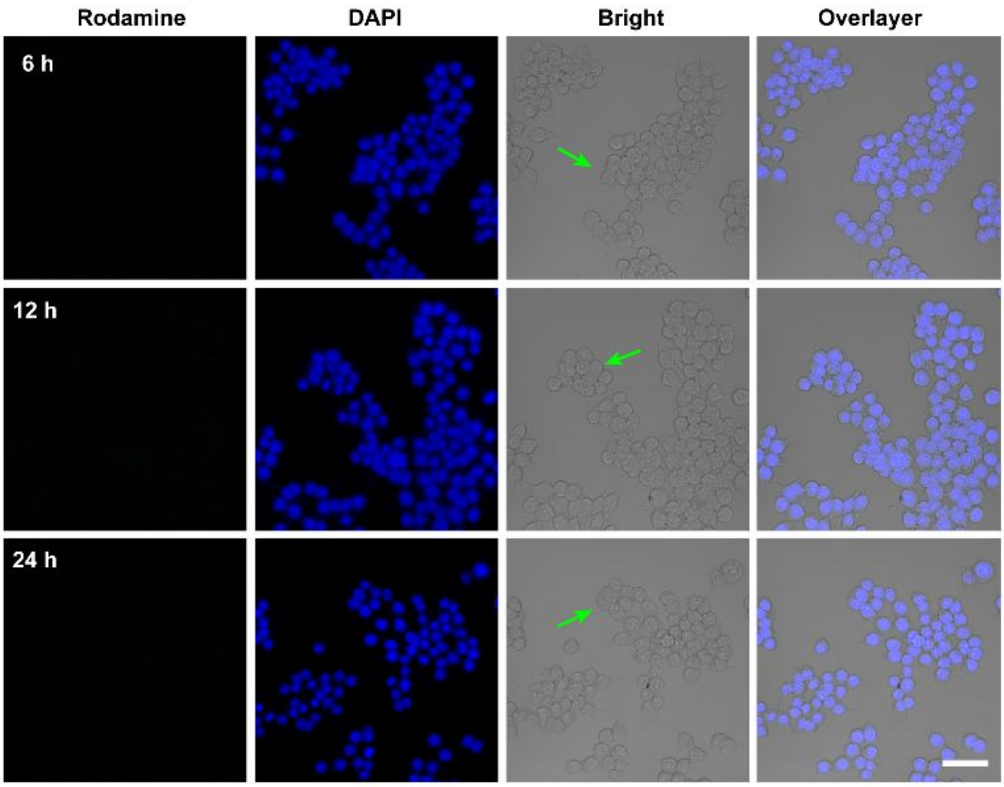

Figure S6. Confocal imaging of RAW 264.7 cells treated with R-BSA at the equivalent concentration for 24 h (scale bar = 40 μm).

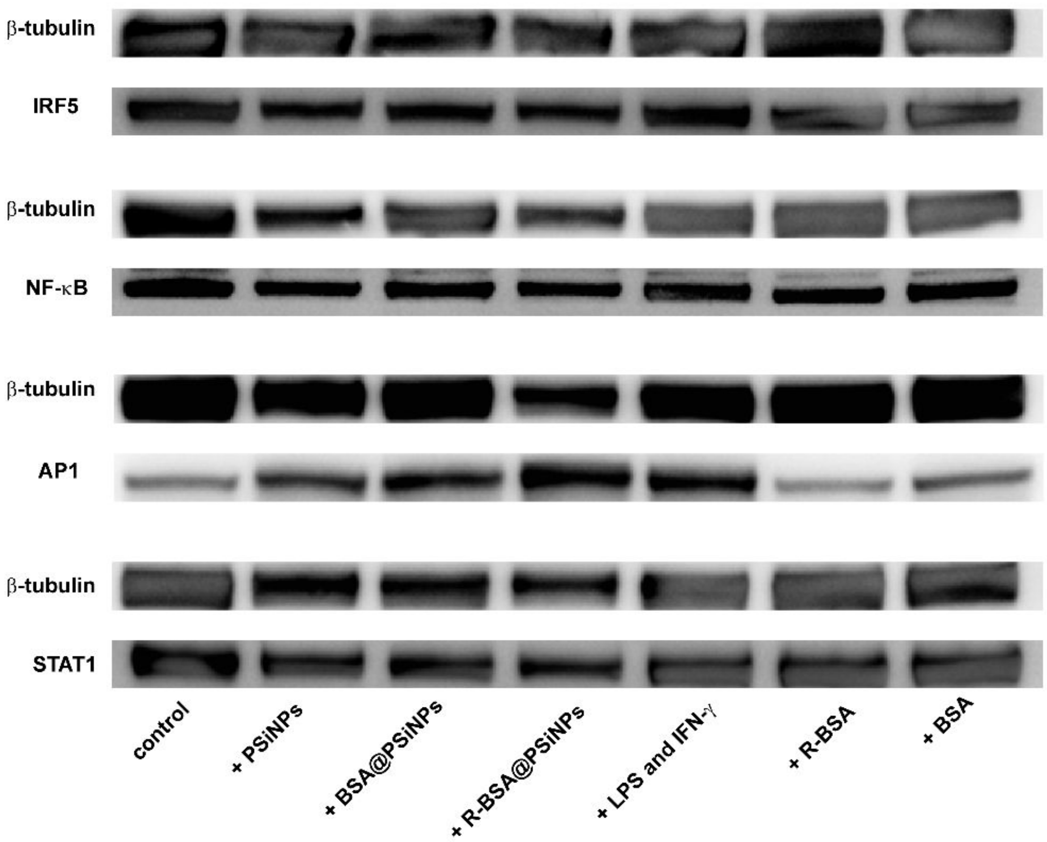

Figure S7. The protein expression levels of RAW 264.7 cells after 24 h treatment with LPS + IFN-γ, 100 μg/mL R-BSA@PSiNPs, 100 μg/mL BSA@PSiNPs, 100 μg/mL PSiNPs, and BSA/R-BSA at the equivalent concentration (β-tubulin as an internal reference for quantitative analysis).

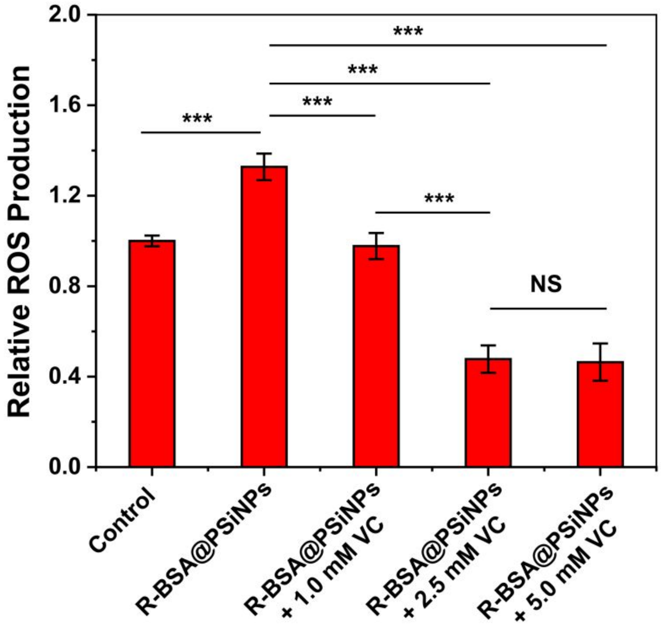

Figure S8. ROS production of RAW 264.7 cells incubated with R-BSA@PSiNPs (100 μg/mL), R-BSA@PSiNPs (100 μg/mL) + VC (1.0 μM), R-BSA@PSiNPs (100 μg/mL) + VC (2.5 μM), or R-BSA@PSiNPs (100 μg/mL) + VC (5.0 μM) for 24 h, respectively, and untreated cells as a control (*n* = 4 biological independent samples). Error bars are based on standard errors of the mean (***P < 0.001, NS > 0.05 by ANOVA with Tukey's post-test).

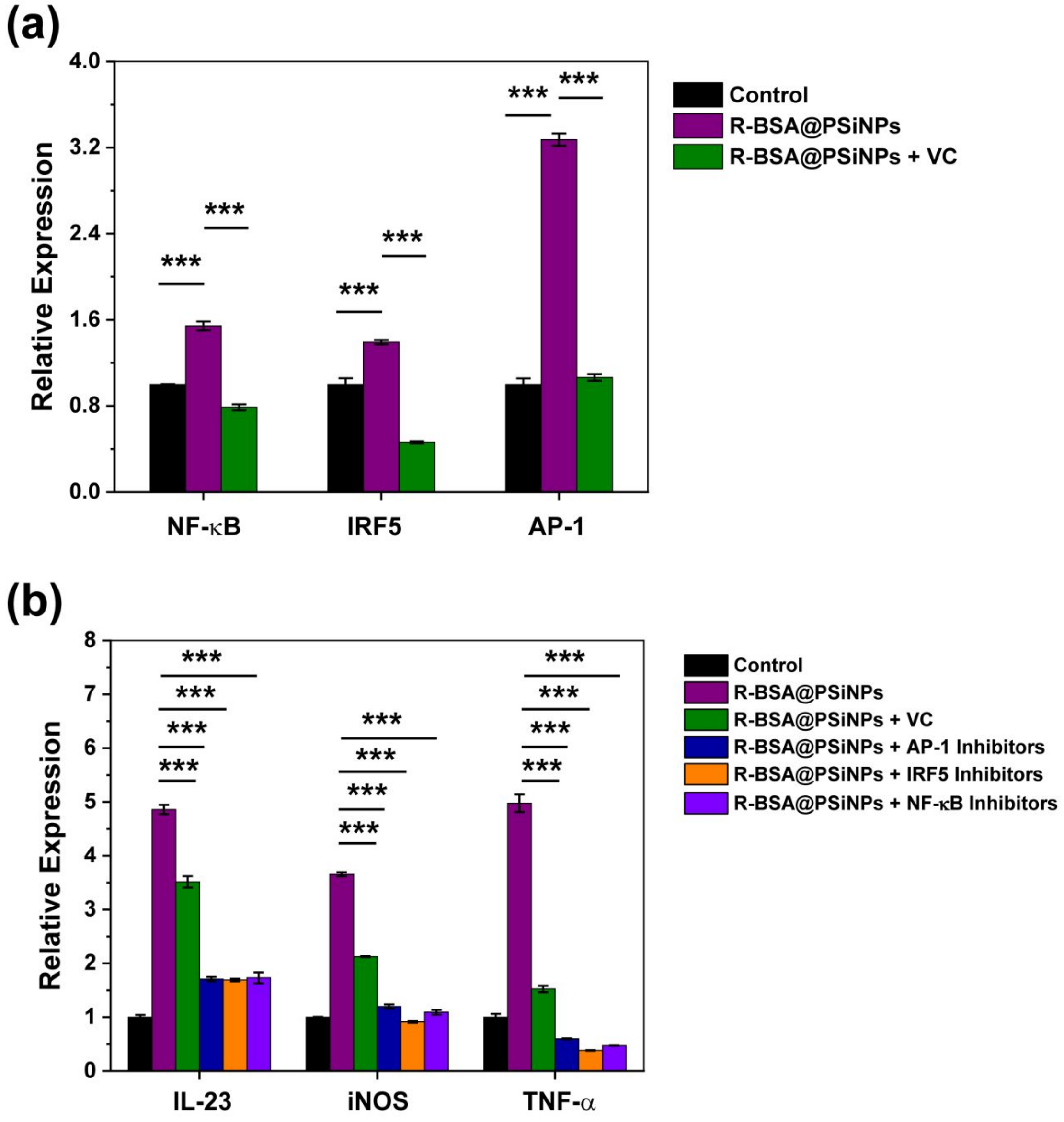

Figure S9. (a) Gene expression of NF-κB, IRF5, and AP-1 in RAW 264.7 cells treated with 100 μg/mL R-BSA@PSiNPs, or 100 μg/mL R-BSA@PSiNPs + 2.5 μM VC for 24 h; and (b) gene expression of iNOS, TNF-α, and IL-23 in RAW 264.7 cells treated with 100 μg/mL R-BSA@PSiNPs, 100 μg/mL R-BSA@PSiNPs + 2.5 μM VC, 100 μg/mL R-BSA@PSiNPs + 20 μM NF-κB inhibitor, 100 μg/mL R-BSA@PSiNPs + 20 μM AP-1 inhibitor, and 100 μg/mL R-BSA@PSiNPs + 1 μM IRF5 inhibitor for 24 h. Untreated cells as a control (*n* = 3 biological independent samples). Error bars are based on standard errors of the mean (****P* < 0.001 by ANOVA with Tukey's post-test).

Materials	XPS Results (%, wt)				DLS Results		
	C 1s (284.5 eV)	O 1s (533.0 eV)	N 1s (399.9 ev)	Si 2p (103.2 eV)	Size (nm)	Zeta Potential (mV)	PDI
PSiNPs	45.10	24.42	0.00	30.46	266.7 ±2.55	-29.1 ±0.60	0.15 ±0.01
R-BSA@PSiNPs	56.01	29.80	4.12	10.05	292.2 ±2.55	-2.04 ±0.12	0.15 ±0.02

Table S1. The quantitative results of XPS and DLS measurements of PSiNPs and R-BSA@PSiNPs nanocomposites.
